# Phylogeography of the Spanish Moon Moth *Graellsia isabellae* (Lepidoptera, Saturniidae)

**DOI:** 10.1186/s12862-016-0708-y

**Published:** 2016-06-24

**Authors:** Neus Marí-Mena, Carlos Lopez-Vaamonde, Horacio Naveira, Marie-Anne Auger-Rozenberg, Marta Vila

**Affiliations:** Department of Molecular and Cell Biology, Evolutionary Biology Group (GIBE), Universidade da Coruña, A Fraga 10, E-15008 A Coruña, Spain; AllGenetics & Biology, SL, Edificio de Servizos Centrais de Investigación, Campus de Elviña, E-15008 A Coruña, Spain; INRA, UR633 Zoologie Forestière, F-45075 Orléans, France; IRBI, UMR 7261, CNRS/Université François-Rabelais de Tours, 37200 Tours, France

**Keywords:** *COI*, Microsatellites, cpDNA, *Pinus sylvestris*, *Pinus nigra*, Mito-nuclear discordance

## Abstract

**Background:**

Geographic and demographic factors as well as specialisation to a new host-plant may lead to host-associated differentiation in plant-feeding insects. We explored the phylogeography of a protected moth, *Graellsia isabellae*, and its two recognised host-plant species (*Pinus sylvestris* and *P. nigra*) in order to seek for any concordance useful to disentangle the evolutionary history of this iconic lepidopteran.

**Results:**

DNA variation in one mitochondrial marker and nine nuclear microsatellite loci revealed a strong phylogeographic pattern across 28 populations of *G. isabellae* studied in Spain and France comprising six groups mostly distributed along different mountain ranges. Reanalysis of a previously published chloroplast microsatellite dataset revealed a three and two-group structure for Spanish *P. sylvestris* and *P. nigra*, respectively. Overall, the population groupings of this protected moth did not match the ones of *P. sylvestris* and *P. nigra*.

**Conclusions:**

There was no evidence of host-associated differentiation between populations using *P. sylvestris* and the ones inhabiting *P. nigra*. The two major mitochondrial clades of *G. isabellae* likely diverged before the Last Glacial Maximum and geographically separated the species into a “southern” (Central and Southern Iberian clusters) and a “northern” lineage (Eastern Iberian, Pyrenean and French Alpine clusters). The Eastern Iberian System, where this insect uses both host-plants, harboured the highest level of genetic diversity. Such a group independently colonised the West and East parts of the Pyrenees. Our results point to a native origin for the French populations occurring in the Alps, genetically related to the Eastern Iberian and Pyrenean sites. The Central Iberian group derived from Southern Iberian ancestors. Secondary contacts were inferred between the Southern/Central Iberian populations and Eastern Iberian cluster as well as between the two Pyrenean ones. The mito-nuclear discordance observed with regard to the Eastern Iberian cluster is congruent with a secondary contact after the evolution of mito-nuclear incompatibilities in geographically isolated areas.

**Electronic supplementary material:**

The online version of this article (doi:10.1186/s12862-016-0708-y) contains supplementary material, which is available to authorized users.

## Background

The diversity of plant-feeding insects is remarkable and host-associated differentiation has been advocated as a relevant trigger of such macroevolutionary diversification [1, 2]. However, not only specialisation to different hosts but genetic incompatibilities between geographically isolated populations may prevent successful hybridisation after a secondary contact [3]. At microevolutionary scale, knowledge about the relative roles played by specialisation to a given host and differentiation in separate areas is also valuable to account for the divergent lineages of oligophagous species of insects [4–6].

**Fig. 1:**
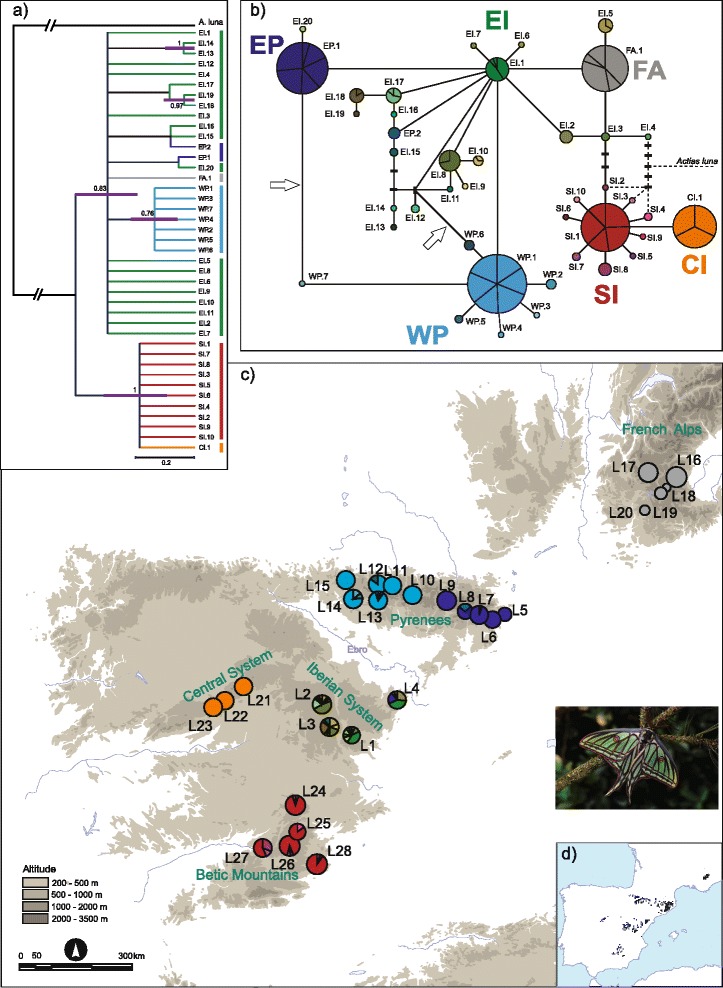
Evolutionary relationships and geographical distribution of mtDNA *COI* haplotypes of *Graellsia isabellae*. The six clusters are displayed in different colours. Tones of dark and light blue refer to haplotypes from WP and EP, whereas tones of green and red correspond to EI and SI. **a** Bayesian phylogenetic tree. Violet bars at supported nodes indicate the temporal estimates (95 % HPD intervals); time scale indicates 0.2 Mya; numbers above branches represent posterior probabilities higher than 0.7. **b** 95 % Statistical Parsimony network, intraspecific connection limit = 12. *Actias luna* was equally connected to haplotypes EI.4, SI.2, SI, 3 and SI.4 by 66 mutational steps. Circle size is proportional to haplotype frequency. Names besides circles are haplotype codes. Solid lines connecting haplotypes represent a single mutational event, regardless of their length. Black rectangles represent missing or theoretical haplotypes. Arrows point to the most likely break of a loop according to coalescent predictions. **c** Geographical distribution of the 41 mitochondrial haplotypes in 28 populations across its entire distribution range. Population codes as labelled in Table 1. Inset: Adult male of *G. isabellae*. **d** Approximate geographic distribution of *G. isabellae* (redrawn from [67, 99])

Here we set out to disentangle the microevolutionary history of the Spanish Moon Moth *Graellsia isabellae* (Graells 1849) (Lepidoptera, Saturniidae), a protected species in France and Spain after its inclusion in the Habitats Directive [7, 8]. This univoltine insect is mainly distributed in the mountains of the eastern half of the Iberian Peninsula and the French Alps (Fig. 1d) and it develops on two cold-adapted host-plants: the Scots (*Pinus sylvestris*) and Black (*P. nigra*) pines. *P. sylvestris* is the host species of *G. isabellae* in the Central Iberian System, Pyrenees and Alps, where the presence of *P. nigra* is scarcer. This spectacular silkmoth inhabits forests of both *P. sylvestris* and *P. nigra* in the Iberian System [9–11]. Although it is widely accepted that *G. isabellae* feeds exclusively on *P. nigra* in Southern Spain [12], the host-plant of the recently discovered population of Sierra-María (*L28*, Fig. 1), flying in a mixed forest of the thermophilous *P. halepensis/P. pinaster*, remains to be confirmed [13]. Our aims are to:

1. Assess the levels of genetic variability and population structure of *Graellsia isabellae* across its entire distribution range.
2. Evaluate whether host-associated differentiation and divergence of geographically isolated populations played a relevant role in the phylogeography of this iconic insect.

*Pinus* species are known for their variation in deterrent compounds (e.g. terpenoids) [14]. Thus, it is reasonable to expect populations of *G. isabellae* to have experienced host-associated differentiation. If so, congruence between the population structure of *G. isabellae* and its host plant use (*P. sylvestris*, *P. nigra* and *P. pinaster/P. halepensis*) should be found.

The evolutionary history of *G. isabellae* is expected to depend to some extent on the range shifts suffered by *P. sylvestris* and *P. nigra* during the Upper Quaternary [15]. Several Last Glacial Maximum (LGM) refugia have been inferred for *P. sylvestris* and *P. nigra* in the Iberian Peninsula [16, 17]. Both species expanded during the Holocene, especially *P. sylvestris* [18, 19]. However, the potentially suitable area for *G. isabellae* in Spain is about three times larger than the one it presently occupies [10]. Therefore, another plausible expectation is that the current degree of population structuring of *G. isabellae* was already present at the LGM, corresponding to the separate glacial refugia of *P. sylvestris*/*P. nigra*.

## Methods

To achieve aim 1, we Sanger sequenced the second half of the cytochrome c oxidase subunit I gene (*COI*), a mitochondrial marker widely used in insect population genetics, and genotyped nine microsatellite markers specifically developed for *Graellsia isabellae* [20]. To attain our second aim, we used the genetic structure of *Pinus sylvestris* and *P. nigra* as a background. For this, we revised the available organelle and nuclear evidence for both pines at European level. We also surveyed the structure of their Iberian populations by reanalysing the chloroplast (cpDNA) microsatellite dataset published by Soto et al. [21].

### Non-lethal sampling and DNA extraction

Between 2007 and 2009, 796 adult males of *G. isabellae* were collected from 28 locations, covering the whole distribution range (Table 1, Fig. 1c). Specimens were hand-netted after being attracted by either captive virgin females or by synthetic female pheromone placed near a light trap [22]. Tissue sampling was performed by clipping a ~ 130 mm^2^ fragment of the right hind-wing tail [23]. Genomic DNA was extracted using a commercial kit (High Pure PCR Template Preparation Kit, Roche) following the manufacturer’s instructions.

**Table 1:** Genetic diversity within samples of *Graellsia isabellae* as from one mitochondrial and nine microsatellite markers

				mtDNA						Microsatellites									
Locality	Mountain range	Coordinates	Code	*N*	*S*	*Nh*	*h*	*π*	*k*	*N*	*NA*	*A*_E_	*AR*	*APR*	*H*_o_	*F*_IS_	*H*_e_	HWE	*H*_MD-SMM_
Ademuz	Iberian	40°04′17.4″N	L1	17	10	8	0.824 ± 0.082	0.0028 ± 0.0005	2.324	32	84	6.0	5.2	0.36	0.655	**0.104**	0.731	***	NS
		1°06′19.8″W																	
Bronchales	Iberian	40°31′19.5″N	L2	21	9	8	0.752 ± 0.086	0.0022 ± 0.0004	1.867	31	85	5.4	5.36	0.39	0.675	**0.106**	0.755	NS	NS
		1°38′56.1″W																	
Huerta	Iberian	40°12′15.5″N	L3	20	8	8	0.874 ± 0.041	0.0032 ± 0.0003	2.637	32	93	5.8	5.47	0.36	0.672	**0.116**	0.760	***	NS
		1°41′31.1″W																	
Els Ports	Iberian	40°47′29.9″N	L4	19	4	5	0.743 ± 0.064	0.0012 ± 0.0002	1.029	41	89	5.5	5.29	0.35	0.703	0.061	0.749	NS	NS
		0°18′45.8″E																	
Albanyà	East Pyrenees	42°18′33.4″N	L5	11	0	1	0.000 ± 0.000	0.0000 ± 0.0000	0	11	28	2.3	2.78	0.03	0.444	0.075	0.481	NS	***
		2°42′14.8″E																	
Castellfolit	East Pyrenees	42°13′22.9″N	L6	17	0	1	0.000 ± 0.000	0.0000 ± 0.0000	0	18	27	2.2	2.66	0	0.377	0.207	0.475	***	***
		2°32′40.1″E																	
Montesquiu	East Pyrenees	42°14′14.6″N	L7	20	1	2	0.100 ± 0.088	0.0001 ± 0.0001	0.100	57	37	2.3	2.85	0.01	0.432	**0.123**	0.493	***	*
		2°21′14.4″E																	
Montgrony	East Pyrenees	42°16′11.9″N	L8	14	2	2	0.440 ± 0.112	0.0011 ± 0.0003	0.879	45	36	2.3	2.98	0.02	0.449	**0.139**	0.521	***	***
		2°05′00.5″E																	
Baiasca	East Pyrenees	42°30′07.2″N	L9	22	0	1	0.000 ± 0.000	0.0000 ± 0.0000	0	25	32	2.3	2.86	0.01	0.488	−0.061	0.460	NS	**
		1°08′59.0″E																	
Renanué	West Pyrenees	42°29′31.7″N	L10	20	0	1	0.000 ± 0.000	0.0000 ± 0.0000	0	31	37	2.7	3.23	0.01	0.504	0.074	0.544	NS	**
		0°31′42.6″E																	
La Sarra	West Pyrenees	42°38′23.7″N	L11	19	0	1	0.000 ± 0.000	0.0000 ± 0.0000	0	23	37	2.9	3.29	0.01	0.560	−0.000	0.560	NS	*
		0°09′13.1″E																	
Ordesa	West Pyrenees	42°39′16.7″N	L12	20	1	2	0.268 ± 0.113	0.0003 ± 0.0001	0.268	27	34	2.7	3.05	0.05	0.482	0.112	0.542	NS	**
		0°04′49.6″W																	
Cabas	West Pyrenees	42°26′06.1″N	L13	20	3	4	0.284 ± 0.128	0.0004 ± 0.0001	0.300	32	40	2.9	3.23	0.05	0.510	0.066	0.547	NS	***
		0°10′16.7″W																	
San Juan	West Pyrenees	42°30′59.9″N	L14	21	2	3	0.410 ± 0.121	0.0005 ± 0.0002	0.438	24	40	3.1	3.24	0.06	0.472	0.149	0.551	***	***
		0°41′26.2″W																	
Belabarce	West Pyrenees	42°52′38.4″N	L15	20	0	1	0.000 ± 0.000	0.0000 ± 0.0000	0	31	30	2.4	2.83	0.02	0.459	0.102	0.511	***	***
		0°53′12.9″W																	
Ange Gardien	French Alps	44°44′14.6″N	L16	25	0	1	0.000 ± 0.000	0.0000 ± 0.0000	0	40	17	1.2	1.49	0.03	0.128	0.054	0.135	NS	***
		6°46′8.31″E																	
Fournel	French Alps	44°45′55.3″N	L17	22	0	1	0.000 ± 0.000	0.0000 ± 0.0000	0	36	15	1.2	1.48	0	0.133	0.151	0.156	NS	***
		6°31′51.4″E																	
Cristillan	French Alps	44°40′29.0″N	L18	4	0	1	0.000 ± 0.000	0.0000 ± 0.0000	0	12	13	1.2	1.38	0	0.120	0.047	0.126	NS	***
		6°42′02.2″E																	
Guillestre	French Alps	44°42′19.8″N	L19	9	0	1	0.000 ± 0.000	0.0000 ± 0.0000	0	11	15	1.4	1.59	0	0.111	0.415	0.190	NS	***
		6°43′24.2″E																	
Auzet	French Alps	44°16′55.6″N	L20	6	0	1	0.000 ± 0.000	0.0000 ± 0.0000	0	6	13	1.3	1.44	0	0.241	−0.413	0.170	NS	***
		6°17′52.6″E																	
Rascafría	Central Iberian	40°51′24.7″N	L21	19	0	1	0.000 ± 0.000	0.0000 ± 0.0000	0	23	22	1.9	2.21	0.11	0.353	0.114	0.398	NS	**
		3°53′31.6″W																	
Cercedilla	Central Iberian	40°45′28.5″N	L22	19	0	1	0.000 ± 0.000	0.0000 ± 0.0000	0	25	27	1.9	2.35	0	0.369	0.077	0.390	NS	*
		4°04′17.7″W																	
Peguerinos	Central Iberian	40°40′57.1″N	L23	20	0	1	0.000 ± 0.000	0.0000 ± 0.0000	0	48	25	1.8	2.25	0	0.305	**0.209**	0.386	NS	**
		4°10′40.9″W																	
Río Mundo	Betic Mountains	38°27′21.3″N	L24	19	2	3	0.205 ± 0.119	0.0003 ± 0.0002	0.211	22	49	3.8	3.81	0.09	0.485	0.166	0.581	***	NS
		2°26′18.2″W																	
Guillimona	Betic Mountains	38°03′16.5″N	L25	13	0	1	0.000 ± 0.000	0.0000 ± 0.0000	0	13	45	4.1	3.95	0.04	0.556	0.031	0.573	NS	NS
		2°33′12.0″W																	
Sagra	Betic Mountains	37°56′05.2″N	L26	20	1	3	0.195 ± 0.115	0.0002 ± 0.0002	0.200	35	62	4.0	4.12	0.1	0.545	0.080	0.592	NS	*
		2°35′25.5″W																	
Cazorla	Betic Mountains	37°54′07.0″N	L27	16	2	3	0.608 ± 0.090	0.0008 ± 0.0002	0.692	34	58	4.0	4.05	0.16	0.542	0.086	0.592	NS	NS
		2°56′18.4″W																	
Sierra María	Betic Mountains	37°44′01.9″N	L28	20	2	3	0.195 ± 0.115	0.0002 ± 0.0002	0.200	31	38	2.3	2.98	0.35	0.433	0.105	0.484	***	**
		2°01′18.7″W																	
Global				493	33	41	0.868 ± 0.006	0.0044 ± 0.0001	3.650	796									

mtDNA: *N* sample size, *S* segregating sites, *Nh* number of haplotypes, *h* haplotype diversity, *π* nucleotide diversity, *k* average number of nucleotide differences

Microsatellites: *N* sample size, *NA* number of alleles per populations, *A* mean of alleles per population, *AR* allelic richness, *APR* private allelic richness, *H*_o_ observed heterozygosity, *H*_e_ expected heterozygosity, *HWE* deviations from Hardy-Weinberg equilibrium, *** significant after sequential Bonferroni correction (*p* < 0.0017), *F*_IS_ = inbreeding coefficient (significant values, *p* < 0.0002, are shown in bold), *H*_MD_ = expected heterozygosity at mutation-drift equilibrium under the SMM model: *p < 0.05, **p < 0.01, ***p < 0.001

### Mitochondrial DNA

We sequenced the 3′ end (832 bp) of the mitochondrial cytochrome c oxidase subunit I (*COI*) gene for 493 of the 796 sampled individuals. Amplifications were carried out in 30 μL volumes containing 1X PCR Buffer (5 PRIME), 1.5 mM MgCl_2_, 1U *Taq*DNA Polymerase (5 PRIME), 0.2 mM of each dNTP, 0.2 μM of each primer C1-J-1751 and C2-N-3661 [24] and 30 ng of DNA. Reaction conditions consisted of 2 min 95 °C followed by 30 cycles of 1 min 94 °C; 1 min 57 °C; 1.5 min 68 °C and, lastly, a final extension of (7 min 68 °C). Amplified fragments were purified and bidirectionally sequenced at DNA Sequencing Service (Macrogen, Korea) using the following primers: ISAF (5′-GGTGACCCAATTCTTTACCAAC-3′, present work, positions 12,811–13,642 of the *Bombyx mandarina* mitochondrion genome), LepLEUr [25]. Inspection of electropherograms and alignmentents were performed in CODONCODES 3.7.1.1 (CodonCode, USA).

### Microsatellite genotyping

The 796 samples were genotyped at ten microsatellite loci previously developed [20]. PCR products (1.2 μL) were mixed with 16 μL formamide containing GENESCAN-500 (ROX) Size Standard (Applied Biosystems, ABI) and the allele size of PCR products was determined on a 96-capillary 3730*xl* DNA Analyzer (ABI). Allelic peaks were independently called by two researchers, using GENEMAPPER 4.0 (ABI). Results from locus *GI03* were not included in the present study due to its complex allelic pattern.

### Genetic diversity and population structure

Nucleotide and haplotype diversities were analysed for the mitochondrial *COI* dataset with DNASP 5.1 [26]. Nuclear gene diversity and *F*_IS_ values for each sampling location were calculated with FSTAT 2.9.3.2 [27]. Allelic richness and allelic private richness were obtained using HP-RARE [28]. Analyses of Hardy-Weinberg equilibrium (HWE) as well as tests for gametic phase disequilibrium were computed using the web version of GENEPOP 4.2 [29]. Genetic differentiation between populations was estimated using pairwise *Φ*_ST_ and *F*_ST_ as implemented in ARLEQUIN 3.5.1.2 [30].

We explored global genetic structure using both the mitochondrial and nuclear datasets. We applied the Spatial Analysis of Molecular Variance (SAMOVA 1.0) [31] to the former by using 100 simulated annealing processes for *K* values (groups of populations) from two to 10. Each run was repeated three times to check for consistency. In addition, we carried out population mixture analyses using the spatial clustering modules implemented for DNA sequence data in BAPS 6.0 [32, 33], both for individuals and groups of individuals (sampling localities). We ran the program ten times each, using *K* = 10, *K* = 15 and *K* = 20 as upper bounds to the number of populations. In each repetition the program explores the space of partitions and informs of the optimal one (highest log marginal likelihood value).

Two Bayesian clustering algorithms were used to infer population structure from the microsatellite dataset: STRUCTURE 2.3.4 [34] and BAPS 6.0. The former was run under the admixture model with correlated allele frequencies between populations [35]. We set a burn-in of 100,000 iterations followed by 500,000 iterations for parameter estimation. Each simulation was run 20 times, exploring values for *K* ranging from one to 29. We chose the best partition of the data by examining both the log probability of the data (ln Pr(*X*|*K*)) and the *ΔK* statistic, following Evanno et al. [36]. CLUMPP 1.1.2 [37] was used to permute the admixture coefficients for the several independent runs resulting for the chosen *K*-value. Finally, DISTRUCT 1.1 [38] was employed to visualize the output from CLUMPP. Multilocus genotypes were clustered using BAPS by grouping individuals (genetic mixture analysis) under a non-spatial model. We set 10 repetitions of the algorithm for each *K* ranging between 1 and 28. The best partition (optimal *K*) was identified as explained above.

In addition, Cavalli-Sforza and Edward’s chord distances (*D*_C_) were obtained with the aid of MICROSAT 1.5 [39] which were used to build Neighbor-joining (NJ) trees with NEIGHBOUR. Confidence in tree topology was assessed by bootstrapping over loci (10,000 samples), and resulting trees were summarized by CONSENSUS. Both programs are implemented in the PHYLIP 3.68 package [40].

### Phylogenetic analyses of mitochondrial sequences

A 95 % Statistical Parsimony (SP) network was calculated for the *COI* haplotypes using TCS 1.2 [41]. Preliminary analyses using the homologous sequence from related species *Actias luna*, *A. selene* and/or *Argema mittrei* (KX302402, JX186589 and KX348010, respectively), revealed *Actias luna* (Linnaeus 1758) as an appropriate outgroup. In order to identify the earliest diverging haplotype/s, maximum connection limit was set to 70. Mitochondrial phylogenetic trees were reconstructed using Bayesian inference, with the aid of MRBAYES 3.2 [42], specifying a partitioned analysis by separating 1^st^ and 3^rd^ codon positions into different partitions, and eliminating the 2^nd^ position of the *COI* alignment due to its lack of phylogenetic information (it only shows a single private change). We used a gamma model of rate variation across sites and sampled across the GTR model space in the Bayesian Markov chain Monte Carlo (MCMC) analysis itself [43], running two simultaneous, independent runs during 10^6^ generations, with trees sampled every 10^3^ generations. The analysis was carried out with default priors until the standard deviation of split frequencies dropped below 0.01, and the potential scale reduction factor for all parameters lay close to 1.0. Two completely independent analyses starting from different random trees were run. For the MCMC sampling of the target distribution, three heated chains and one cold chain were used. The first 25 % samples of the cold chain were discarded as burn-in. We used Bayes factor comparisons to test several topological hypotheses. Marginal model likelihoods were estimated by the stepping-stone method; strength of the evidence in favour of the better model was then assessed by the magnitude of the log-difference, following Kass & Raftery [44]. The strict clock model was tested against the non-clock model in this way, and once determined that our dataset evolved in a clock-like manner, we obtained a calibrated tree using the rate dating approach, based on the divergence rate of 3.54 % My^−1^ estimated for the *COI* gene in tenebrionid beetles [45], essentially due to changes in 1^st^ or 3^rd^ coding positions. The output cladogram summarizing the trees was visualized with FIGTREE 1.4.2 [46], retaining branch length information and expressing nodal support as posterior probabilities. Mean ages and 95 % highest posterior density (HPD) intervals of mtDNA phylogroups are used as estimates of divergence times.

### Historical demography

We investigated the effects of fluctuations in population size on the genetic variation of the population clusters defined in this work. On the one hand, we used the *COI* dataset to plot the observed frequency distribution of pairwise nucleotide differences with their expected distribution by performing a mismatch analysis. We estimated the demographic parameters θ_0_, θ_1_ and τ from the mismatch distribution using MISMATCHHETMUTRATE [47], based on a finite-sites model with heterogeneity of mutation rates; confidence intervals around the estimated parameters were obtained by bootstrapping (1000 samples). Goodness of fit to the expected mismatch distribution under different theoretical demographic scenarios was tested using the sum of squared deviations (SSD), by comparing observed values with those yielded by 1000 simulated populations. To obtain an approximate time for the putative expansion events, we followed [48], so that *t* = τ/2*u*; *u* = μ*Lg*, where μ is the estimated mutation rate (0.0177 substitutions/site/my), *L* is the length of the sequence (832 bp), and *g* is the generation time (1 year). In addition, we used Fu’s *F*s and *R*_2_ tests, the more powerful ones for detecting departures from the null hypothesis of constant population size [49, 50]. Confidence intervals and *P*-values of these tests were obtained by Monte Carlo simulations (10,000 pseudo-replicates) carried out with DNASP, based on the neutral coalescent process (random-mating population of constant size, with all mutations selectively neutral and occurring at sites that have not previously mutated) and assuming no recombination. Coalescent simulations for the *R*_2_ statistic were conditioned on the number of segregating sites.

As a separate approach, BOTTLENECK 1.2.02 [51] was used to detect reductions in population size using our microsatellite dataset as follows. Expected heterozygosities at mutation-drift equilibrium (*h*_MD_), obtained from the observed number of alleles at each locus through simulating the coalescent process for *n* loci, are compared to heterozygosities at HWE (*h*_HW_). During a bottleneck the number of alleles decreases faster than gene diversity, and therefore *h*_HW_ should be higher than *h*_MD_. The analyses were performed across 10,000 iterations under both the infinite allele model (IAM) and the stepwise mutation model (SMM), which respectively correspond to the least and most stringent conditions to detect a bottleneck. We assessed the significance of the results using the Wilcoxon’s signed ranks test, implemented in the program. We also investigated the distribution of allele frequencies, checking for the expected distortions (mode shifts) produced by recent bottlenecks [52].

### Phylogeography of *Pinus sylvestris* and *P. nigra* as from published genetic data

With regard to *Pinus sylvestris*, we inspected the mitochondrial results published by Sinclair et al. ([53] haplotypes obtained by RFLP analysis of *COI*), Soranzo et al. [54] and Cheddadi et al. [16], both of whom analysed one intron (*nad1*, exon B/C). Then we surveyed the dataset from Naydenov et al. ([55] haplotypes revealed by the combination of the *nad7* intron 1 and the *nad1* intron B/C) and Pyhäjärvi et al. [56], whose mitochondrial haplotypes were combinations of the ones published by Soranzo et al. [54]. Then, we revised the interpretation of the results about population structure of Iberian Scots pine by Robledo-Arnuncio et al. [57] using chloroplast SSR and Prus-Glowacki et al. [58] based on isoenzyme data.

To the best of our knowledge, the only mitochondrial data from *Pinus nigra* related to forests of South Spain and Morocco [59]. In addition, we also revised the interpretation of the chloroplast SSR results by Afzal-Rafii & Dodd [17] and the genomic ISSR data by Rubio-Moraga et al. [60].

### Genetic structure of *Pinus sylvestris* and *P. nigra* as from cpDNA

We reanalysed the cpDNA SSR dataset published by Soto et al. [21] for Iberian *P. sylvestris* and *P. nigra*. The former consisted of 706 individuals, sampled in 30 natural forests and genotyped for six loci. We excluded locus *PT1254* from our analyses as it contained 20.25 % of missing data. The percentage of missing data for the other five loci ranged between 0.56 (*PT30204*) and 2.12 (*PT1936*). The available cpDNA SSR dataset for *P. nigra* consisted of 326 individuals, sampled in 14 natural forests and genotyped for the same six markers. In this case, all loci were used, as missing data ranged from 0.92 % (*PT30204*) and 3.37 % (*PT1254*). Preliminary runs revealed that the use of missing data would produce slight differences in the clustering pattern of *P. nigra*. Therefore, 30 individuals of Black pine (1–6 individuals per sampling site, 11 localities affected) had to be discarded from the final analysis.

The overall genetic structure of both conifers was assessed using the Bayesian clustering implemented in BAPS 6.0. We applied a non-spatial genetic mixture analysis to groups (sampled localities) of haplotypes and the “linked loci” option. Again, we performed ten repetitions of the algorithm for each *K* ranging between 1 and the number of sampled sites (*P. sylvestris* = 30, *P. nigra* = 14). The best partition (optimal *K*) was identified as explained above.

The population structure of *Pinus nigra* in the South of Spain and Morocco inferred by Jaramillo-Correa et al. [59] was based on the same six markers we reanalysed from Soto et al. [21]. However, the population structure of *P. sylvestris* in the Northern Meseta (NW spain) surveyed by Robledo-Arnuncio et al. [57] relied on the five loci we reanalysed for *P. sylvestris* plus a sixth one, not included in Soto et al. [21]. So, concerning *P. sylvestris*, the only difference in terms of molecular markers is the exclusion of locus *PT1254* in our dataset and the information provided by locus *PT26081* in Robledo-Arnuncio et al. [57]. Lastly, it should be noted that 12 out of the 14 Spanish sites sampled by Prus-Glowacki et al. [58] and all (13) localities surveyed by Robledo-Arnuncio et al. [57] are included in the dataset by Soto et al. [21] we reanalysed.

### Typing for three *Wolbachia* genes

Incompatibility between males from infected populations and females from uninfected ones may lead to asymmetrical gene flow, a phenomenon reported in moths [61]. The mitochondrial and nuclear discordant levels of differentiation between the Pyrenean sites *L9* and *L10* (see Results) might have been caused by reproductive parasites such as *Wolbachia*. We tested for presence of this bacterium in a set of 53 specimens of *Graellsia* following Kodandaramaiah et al. [62] (Additional file 1).

## Results

### Genetic diversity and population structure

Thirty-three polymorphic sites along the 832 bp alignment defined 41 mitochondrial haplotypes with a strong phylogeographic structure (Table 1). There were 15 parsimony informative sites (14 of them with two variants, whereas position 555 of the alignment showed three variants). Twelve out of the 34 detected mutations caused amino acid replacement (Additional file 2).

Haplotypes were not shared with localities from other mountain ranges, with the sole exception of variants EI.1 (mostly distributed in the Iberian System) and EP.1 (mainly occurring at the Eastern Pyrenees). The first one was shared by three localities of the Iberian System (*L1, L3* and *L4*) and Montesquiu (*L7*), whereas EP.1 was present at Els-Port (*L4*) and all the Eastern Pyrenean sites (*L5-L9*) (Additional file 3). The four sites sampled in the Iberian System (*L1-L4*) showed the highest haplotype and nucleotide diversity (Table 1).

**Fig. 2:**
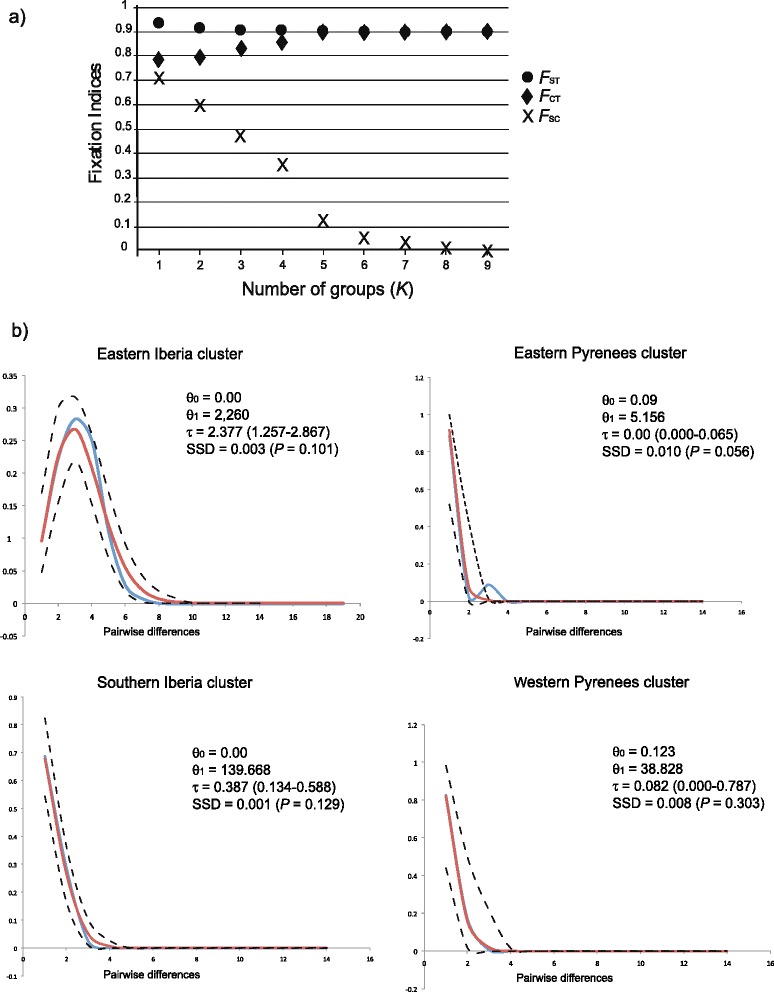
Population structure and demography of *G. isabellae* as from mitochondrial data. **a** Values of fixation indices (*F*_CT_ = among groups differentiation; *F*_SC_ = among populations within groups differentiation; *F*_ST_ = total differentiation among populations) obtained by SAMOVA from a predefined number of groups (*K*) ranging from 1 to 9. **b** Mismatch distributions of polymorphic clusters resulting from STRUCTURE. Model of sudden expansion fit to data. Blue lines show the observed distribution of pairwise nucleotide differences. Red lines were obtained by fitting θ_0_, θ_1_ and τ by using the method of nonlinear least squares. Black dashed lines are the empirical 95 % confidence intervals for the mismatch distribution

Mitochondrial data revealed six groups of populations of *G. isabellae* (SAMOVA, Fig. 2a), which, with one exception (Pyrenees), showed a one to one correspondence with each of the main mountain ranges inhabited by the species: Iberian System (EI: Eastern Iberia, *L1, L2, L3, L4*, in green), Eastern Pyrenees (EP: *L5, L6, L7, L8, L9*, in dark blue), Western Pyrenees (WP: *L10, L11, L12, L13, L14, L15*, in light blue), French Alps (FA: *L16, L17, L18, L19, L20*, in grey), Central Iberian System (CI: Central Iberia, *L21, L22, L23*, in orange), and Betic Mountains (SI: Southern Iberia, *L24, L25, L26, L27, L28*, in red) (Fig. 1c). The spatial clustering of sampling localities calculated in BAPS produced exactly that same optimum partition in all the repetitions of the analysis. At the individual level, though, the most frequently favoured partition (25 out of 30 repetitions) consisted of just five mitochondrial clusters: CI-SI, WP, EP, FA and the EI core. This individual-based analysis assigned some samples from EI to other clusters: most of the specimens from Els-Ports (*L4*) to cluster EP and those specimens from Bronchales and Huerta (*L2* and *L3*) bearing haplotype EI.5 to cluster FA.

Overall, the number of alleles per nuclear marker ranged between six (locus *GI26*) and 37 (*GI11*). Loci *GI18* and *GI23* showed significant departures from HWE in more than two localities. This was likely due to segregating null alleles, as supported by the geographic distribution of these departures, i.e. locus *GI18* deviated from HWE at three sites (*L1-L3*) from the Iberian System and two (*L24, L26*) from the Betic Mountains, whereas HWE was rejected for *GI23* at three localities from the Eastern Pyrenees (*L5-L8*) (Additional file 4). Bayesian clustering and bottleneck analyses were unaffected by their exclusion, so we report nuclear results using all nine microsatellites. No linkage disequilibrium was observed for any pair of loci after Bonferroni correction.

The four sites sampled at the Iberian System (*L1-L4*) showed the highest levels of gene diversity (*H*_e_ = 0.731–0.760). When it comes to private allelic richness, the southern site of Sierra-María (*L28*) showed a similarly high level to the Iberian System localities (0.35–0.39) (Table 1). Seven of the 28 populations were significantly differentiated from all the others as from pairwise *F*_ST_ distances. Five of them are in the Pyrenees (*L9*, *L10*, *L11*, *L14* and *L15*), one in the Central Iberian System (*L21*) and the last one in the Betic Mountains (*L28*). The Western Pyrenean sites (*L10-L15*) revealed a particular substructure, as all pairwise comparisons but one (*L12-L13*) were significant (Additional file 5).

**Fig. 3:**
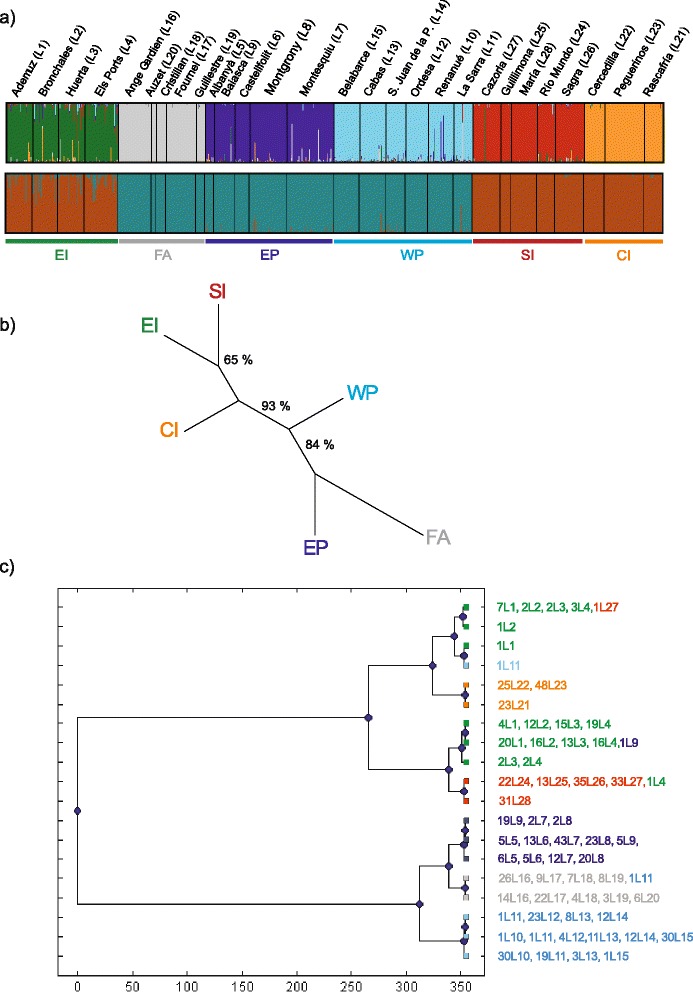
Population structure and phylogeny of *G. isabellae* as from nuclear microsatellites. **a** Bayesian assignment probabilities for *K* = 2 and *K* = 6 revealed by STRUCTURE. Each vertical bar corresponds to one individual. Each background represents the proportion of membership of individuals to the inferred hypothetical groups of populations. Names above the plot represent population codes (Table 1). **b** Unrooted NJ tree of clusters based on *D*_C_ chord distance. Percentages correspond to the bootstrap support values. **c** UPGMA tree based on the estimated Kullback–Leibler divergence between the 19 homogeneous groups obtained by a non-spatial Bayesian cluster analysis implemented in BAPS. Information on the right (#LX) indicates number of individuals (#) from a given site (LX) assigned to that cluster

The most likely partition of the microsatellite dataset was six groups (EI, EP, WP, FA, SI and CI), as from STRUCTURE (Fig. 3a, upper panel, Additional file 6). A second partition (*K* = 2) was supported only by Evanno’s method: the first cluster joined the French Alps, Eastern Pyrenees and Western Pyrenees (FA-EP-WP), whereas the second one grouped Eastern Iberia, Central Iberia, and Southern Iberia (EI-CI-SI) (Fig. 3a, lower panel). However, the overall population structure characterised by BAPS yielded *K* = 19 as the best partition (Fig. 3c). Three of these clusters were formed by a single individual (sampled at Ademuz *L1*, Bronchales *L2* and La Sarra *L11*, respectively). Cluster and sampling locality only matched at Rascafría *L21* and Sierra-María *L28*.

### Phylogenetic analyses

The 33 polymorphic sites observed along the *COI* alignment produced a rather shallow haplotype phylogeny, with many polytomies. Both in the phylogenetic trees (Fig. 1a) and the statistical parsimony network (Fig. 1b) of the *COI* data, the EI cluster joined to FA, EP and WP. These two haplogroups (EI-FA-EP-WP and CI-SI) differ by three mutational steps. Although the first group was not as well supported in the Bayesian trees (posterior probability = 0.83) as the second one (posterior probability = 1), the monophyletic relationship between them was strongly preferred by the Bayes factor test (log difference = 21 log units). The clock-wise evolution of these sequences was also very well supported by the corresponding test (log difference of 35 log units in favour of the strict clock against the non-clock model). The split between these groups would have taken place ca. 400 kya (95 % HPD interval: 270-580 kya), with the most recent common ancestor (MRCA) of EI-FA-EP-WP dated at 300 kya (95 % HPD: 195–405) and that of CI-SI at 190 kya (95 % HPD: 95–310).

Clusters CI, SI, FA, EP and WP generally showed either a single haplotype or a highly predominant one with others arising from it by a single mutational step (Fig. 1b). The only exception to this rule was haplotype EP.2, found at the Eastern Pyrenean locality of Montgrony (*L8*), phylogenetically closer to the haplotypes of the Iberian System (EI cluster, Fig. 1b). The observations of either a single haplotype (CI, FA) or a star-like phylogeny (EP, WP, SI) stand in sharp contrast with the rich diversity and highly reticulated pattern displayed by the haplotypes of the EI cluster. Indeed, the only two terminal variants “geographically misplaced” were part of the EI cluster: EI.20 was found at the northernmost locality of the Eastern Iberian System (*L4*) but differed by a single mutational step from the dominant haplotype at the Eastern Pyrenees (EP.1), whereas EI.5 was sampled at *L2* and *L3* and also differed from the French haplotype (FA.1) by one substitution. Two more features of the mitochondrial network should be pointed out. Firstly, the only haplotype found at the Central Iberian System (CI.1) derives directly from the dominant haplotype at the Betic Mountains (SI.1), in agreement with their joining in a monophyletic group by the Bayesian tree reconstruction. Secondly, the also unique haplotype of the French Alps (FA.1) derives from haplotypes of the Iberian System (cluster EI), not from those of the geographically intervening Pyrenees (clusters EP and WP). Turning again to the phylogenetic tree for corroborative evidence, we used a Bayes factor test to contrast the hypothesis that FA and EI form a group with the hypothesis that EI forms a group with the populations from the Pyrenees (EP and WP) instead, obtaining a strong preference (5 log units) for the former topology.

As from the nuclear perspective, the NJ tree based on average microsatellite chord distances (*D*_*C*_) between clusters showed the highest bootstrap support (93 %) for the branch separating EI-CI-SI from FA-EP-WP. The same partition was first unveiled by STRUCTURE (if *K* = 2) and recovered by BAPS (Fig. 3) and reveals a mito-nuclear discordance with regard to cluster EI. It also presented a noteworthy support (84 % bootstrap value) for the branch joining FA with EP, which makes considerable sense on geographical terms (Fig. 3b).

### Historical demography

Clusters EI, SI and WP showed genetic signatures of population expansions, as revealed by the analysis of their mismatch distributions of mitochondrial haplotypes (Fig. 2a) and departures from constant population size models (Table 2). Based on τ estimates (Fig. 2b), the time since expansion for the EI, SI, and WP clusters would be 81 kya (43-97 kya), 13 kya (4.5-20 kya) and 2.8 kya (0-27 kya), respectively. The very low (EP) or downright absent (FA, CI) mitochondrial diversity observed in the three other clusters makes this kind of analysis inapplicable to them.

**Table 2:** Signatures of population size changes on mitochondrial variation

mtDNA				
Cluster	*N*	*Nh*	Fu’s *Fs*	*R2*
EI	77	21	−12.223***	0.055
EP	84	3	−1.095	0.051
WP	120	7	−8.346***	0.028**
FA	66	1	n.a.	n.a.
SI	88	10	−11.486***	0.030**
CI	58	1	n.a.	n.a.

*N* number of individuals, *Nh* number of haplotypes, *p < 0.05, **p < 0.01, ***p < 0.001

Sites from all clusters but EI likely suffered population size reductions in the near past, as revealed by the comparison of present gene diversity (*H*_HW_) with its expected value in mutation-drift equilibrium (*H*_MD_, conditioned on the number of observed alleles) (Table 1). We conservatively interpreted the results as indicative of bottleneck if the null hypothesis was rejected both under the IAM (all significant, data not shown) and SMM (Table 1) scenarios. The distributions of allele frequencies showed the characteristic L-shape of non-bottlenecked populations in the four localities sampled at EI, as well as the Eastern Pyrenean site of Montesquiu, the Central Iberian site of Peguerinos and the Western Pyrenean locality of Sarra.

### Review on the phylogeography of *Pinus sylvestris* and *P. nigra*

The distribution area of *Graellsia isabellae* somehow resembles the one of *Pinus nigra salzmannii* [15, 63]. The few molecular data available show a strong population differentiation of the Black pine, not only at European level but also between the Northern and Southern Spanish populations [17]. Indeed, mitochondrial data revealed a particular phylogeographic pattern in South Spain, as the Betic Mountains harboured a genetic break, also shared by other conifers [59]. Lastly, nuclear ISSR markers confirmed the existence of population structure within the Betic Mountains as well as the affinity of the Moroccan and Southern Spanish populations found by Jaramillo-Correa et al. [59]. In addition, ISSR markers revealed a much deeper break between these populations and the three sites from Iberian System [60]. More details available at Additional file 7.

Both chloroplast [16] and nuclear data [64–66] indicated the differentiation of Spanish *Pinus sylvestris* when compared to other European populations. Within the Iberian Peninsula, the mitochondrial evidence revealed a particular differentiation of Sierra Nevada, one of the two extant populations of the Scots pine in Southern Spain. However, the other southern locality of Baza (only 80 km apart) showed similar haplotype frequencies to the Central Iberian System [54–56]. The East–West Iberian differentiation as from haplotype frequencies was noticeable indeed [53–56]. The singularity of the Scots pine populations of the Iberian System gains additional interest because of sharing a haplotype with the Balkans [55]).

### cpDNA structure of *Pinus sylvestris* and *P. nigra*

**Fig 4:**
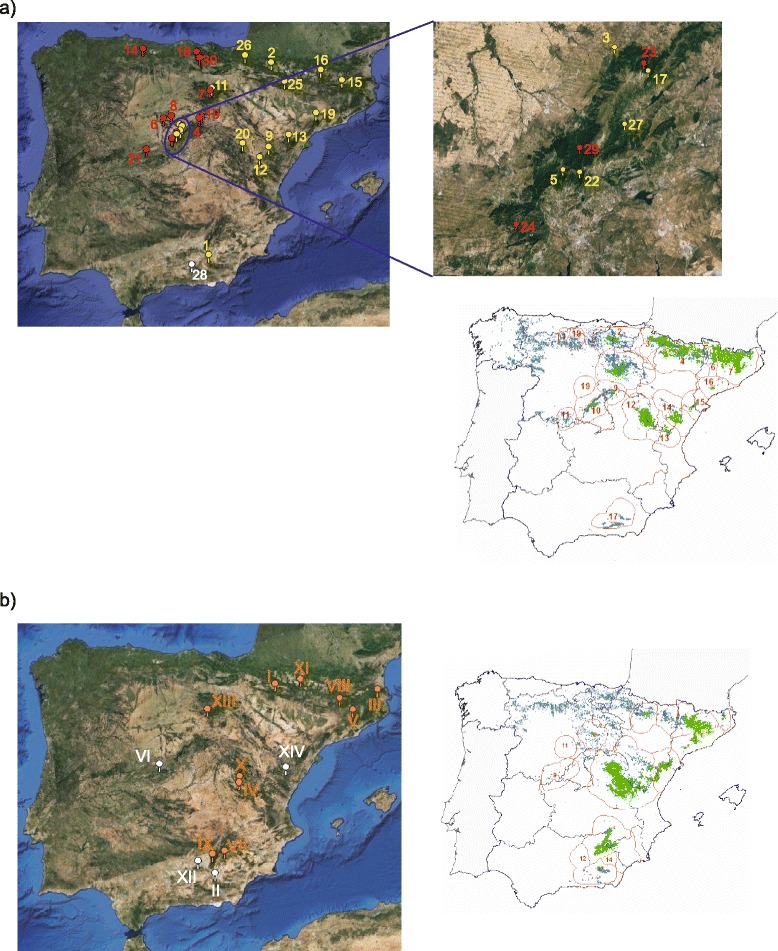
Population structure and distribution area of *Pinus sylvestris* and *P. nigra* in Spain. Results of the non-spatial Bayesian cluster analysis, BAPS, showing genetically homogenous groups of populations of (**a**) *P. sylvestris* and (**b**) *P. nigra* as from the the cpSSR dataset by Soto et al. [21]. Readers are referred to http://www.spatialepidemiology.net/user_maps/php/temp/06-23-15-74566.html and http://www.spatialepidemiology.net/user_maps/php/temp/06-25-15-94314.html for a closer inspection of these maps. Arabic numbers and roman numerals indicate population order as from [21]. Maps on the right [63] show the Spanish distribution range of each species, both natural (*green*) and reforested (*blue*) areas. Red lines and numbers refer to provenances

The overall population structure characterised by BAPS for Iberian *Pinus sylvestris* (five markers) yielded *K* = 2 as the best partition. The southernmost locality (Trevenque, #28, at Sierra Nevada) revealed as the first cluster, whereas the remaining 29 forests grouped together. Removal of this divergent population, led to a two-group partition with a clear West–east orientation (Fig. 4a). Therefore, we can safely infer a three-group population structure of the Spanish Scots pine surveyed by Soto et al. [21].

Concerning *Pinus nigra* (six markers) BAPS indicated again *K* = 2 as the most likely partition. The two southernmost Betic localities (Baza #II, Huelma #XII) grouped with the Central Spanish locality (Casavieja, #VI) and the Eastern Iberian forest coded as #XIV (Vistabella). All other localities clustered together. Such a two-group structure does not match the taxonomic division within *P. nigra salzmannii*, i.e. site #XIV (*pyrenaica*) grouped with the Central Iberian System and the southernmost Betic Mountains, whereas sites #IV and #X (*hispanica*) clustered with the Pyrenees and the northern Betic Mountains (Fig. 4b).

### Typing for three *Wolbachia* genes

We found no evidence of infection by *Wolbachia* in any of the 53 specimens surveyed, 20 of them from *L9* and *L10* (Additional file 1).

## Discussion

### Genetic variability and population structure of *G. isabellae*

*G. isabellae* is structured into two main mitochondrial groups and six nuclear clusters. The mitochondrial “northern lineage” was formed by clusters EI, WP, EP and FA, classified as subspecies *G. isabellae isabellae, G. isabellae roncalensis, G. isabellae paradisea* and *G. isabellae galliaegloria*, respectively (revised by [67]). The EI cluster was the most diverse as measured from mitochondrial and nuclear data. A somehow similar result has been found in the Processionary Pine Moth (site 51 on *Pinus nigra*, [68]). Conversely, FA was the least variable. The mitochondrial “southern lineage” of *G. isabellae* was formed by clusters SI and CI, classified as subspecies *G. i. ceballosi* and *G. i. isabellae*, respectively (revised by [67]). The SI cluster was the second most diverse group in our dataset. Conversely, the CI cluster was mitochondrially monomorphic and its nuclear diversity was intermediate between the FA and EP groups.

The genetic pattern of the EI cluster points to the Iberian System as genetic sanctuary [69] for *G. isabellae*. This hypothesis is congruent with the basal position of haplotype EI.4 and the rich diversity and highly reticulated pattern displayed by the EI haplotypes (Fig. 1b). The ancestral character states of the EI cluster are also supported by the evolutionary history of *P. sylvestris* (Additional file 7).

The reticulated pattern of cluster EI was due to some haplotypes connected to variants from other clusters (WP, EP and FA) as well as to loops caused by ambiguous phylogenetic relationships. Firstly, we argue for incomplete lineage sorting to be the reason why variants EP.2 and EI.5 seem to be geographically “misplaced” if compared to their closest haplotypes (Fig. 1b, Additional file 3). Secondly, recombination and/or homoplasy could account for the twelve EI haplotypes that ambiguously connected to others. These loops were caused by eight third-codon synonymous substitutions (Additional file 2). Distinguishing between recombination after admixture of lineages, paternal leakage and homoplasies caused by reverse or parallel mutation will require larger sample sizes, additional markers as well as other analyses (e.g. [70, 71]. We acknowledge these mechanisms as plausible, as mitochondrial recombination has been reported in other orders of insects [72–74] and mtDNA was paternally inherited in *Antheraea* x *proylei*, an interspecific hybrid from the same family and tribe, Saturniini, as *G. isabellae* [75]. Moreover, the production of new haplotypes by recombination requires heteroplasmy, a phenomenon we observed in two individuals (from France and Switzerland) not included in the present study.

We postulate that cluster WP has been isolated from EI for a longer period than the EP group. Once the coalescent predictions are applied to singletons WP.6 and WP.7 (Fig. 1b), cluster WP becomes the terminal star-like phylogeny typical of a population expansion, occurring at some point in the last 27 kya (Fig. 2b). Such a temporal framework has to be used with caution, both because of the uncertainty about the mutation rate applied and the singular substructure of *G. isabellae* in the Western Pyrenees revealed by the nuclear markers (Additional file 5). Indeed, the relatively wide curve of its mismatch distribution may have been produced not only by the time since expansion but also by the population substructure of the WP cluster [47], which was probably caused by the patchier distribution of *P. sylvestris* in that area (Additional file 8). Nevertheless, the weakly supported WP cluster (Fig. 1a) could have a pre-Holocene origin if genetic drift had shortened the coalescence time of the segregating alleles and produced the accumulation of fixed differences with other clusters. Populations of clusters WP and EP experienced population size fluctuations (Table 1), but our analyses cannot provide information about the timing or strength of those bottlenecks. Thus, we can only speculate about the independent and subsequent action of genetic drift on cluster EP as the main reason of its lower genetic diversity. We base this possibility on the severe fluctuations between arid and humid climatic stages in the Eastern Pyrenees associated with a decreasing genetic diversity from West to East in several Pyrenean plants [76].

We propose a native origin of the French populations (cluster FA) of the Spanish Moon Moth. Some authors considered them to be the result of a deliberate human introduction from Iberian individuals in the early 20^th^ century, e.g. [77, 78]. However, all our evidence indicates that the French Alps were colonised by a small number of founders whose mitochondrial origin can be traced to the Iberian System (EI cluster), probably through an Eastern Pyrenean corridor. Indeed, the mitochondrial haplotype FA.1 was not found in the Iberian populations. FA.1 forms a loop with EI.1 (predominant in the Eastern Iberian System, but present in one individual from Montesquiu), EI.2 (found in five specimens from Els-Ports) and EI.3 (carried by two males from Ademuz). In addition, BAPS grouped the six individuals bearing the EI.5 terminal haplotype with the French specimens. Although we cannot completely rule out a deliberate human introduction from Iberian specimens, the natural foundation of the Alpine ancestral population following the expansions of *P. sylvestris* and/or *P. nigra* during the Holocene is supported by other lines of evidence. Firstly, the level of genetic diversity obtained for the FA cluster is very similar to that found in the Central Iberian System (CI), whose natural origin has never been in dispute. A similar result was obtained for the Northern Pine Processionary Moth (*Thaumetopoea pinivora*) [79]. Secondly, a Holocene spatial migration of some ancestors of the EP cluster northwards following pine expansions is plausible from a biogeographic perspective: (i) suitable habitat (*P. nigra salzmannii*) for *G. isabellae* existed in Southeastern France even before the LGM [80], (ii) the first postglacial expansion of the so-called “light-green haplotype” of *P. sylvestris* was traced (based on macrofossils) from the easternmost part of the Pyrenees northwards [16] and (iii) other montane species dependent on pine forest likely spread from the Eastern Pyrenees northward following Holocene pine expansions [81].

### No host-associated differentiation

The population structure of *G. isabellae* did not support host-associated differentiation as a relevant factor preventing gene flow between populations using *Pinus sylvestris* or *P. nigra*. The indiscriminate use of the Scots and Black pine has been reported in other oligophagous plant-feeding insects [82]. Actually, local adaptations to *P. sylvestris* and *P. nigra* have been reported for the pine processionary moth *Thaumetopoea pityocampa* [83]. Females of this species may recognise the volatiles emitted by different host pine species and exhibit oviposition preference [84], but nevertheless host-associated mitochondrial differentiation was absent [68]. Absence of host-associated (*P. nigra* vs *P. sylvestris*) differentiation has also been reported in *T. pinivora* [85]. Actually, the recent discovery in South Spain of a population of this defoliator that uses *P. nigra* instead of its usual host *P. sylvestris* [85] strikingly resembles the case of *G. isabellae* at Sierra-María, likely using *P. pinaster/P. halepensis* [13]. The distance from Sierra-María to the closest patches of *P. nigra* (28 km North, 36 km West, [63]) makes the attraction and sampling of moths from the few stands of Black pine existing in that area unlikely. If *P. halepensis* (or *P. pinaster*) is finally confirmed as the host-plant of *G. isabellae* at Sierra-María, future research will have to evaluate if its genetic differentiation of this southern locality is just the result of genetic drift (Fig. 3, Additional file 5) or whether adaptive divergence to a new, thermophilous, host is also involved (e.g. [6]).

### *G. isabellae* and the LGM refugia of *Pinus* spp

The five differentiated groups of Spanish *Graellsia isabellae* reside in geographical areas identified as glacial refugia for *Pinus sylvestris* by Cheddadi et al. [16] (SI, EI, WP and EP) and Benito Garzón et al. [18] (the same ones plus CI). The origin of the two major mitochondrial clades observed in *G. isabellae*, the “southern” (CI and SI clusters) and “northern” lineages (EI, EP, WP and FA), fits a two glacial refugia scenario. These two groups would have initiated their divergence approximately 400 kya (Fig. 1a). Regardless of the actual date for their divergence, our data revealed an isolation scenario since, at least, the LGM. This hypothesis will be later revised in the light of the nuclear results (see section *Secondary contacts*), which grouped cluster EI with the so-called “southern lineage”. The differentiated groups obtained for *P. sylvestris* and *P. nigra* (Fig. 4) probably remained isolated since the LGM as well. The deeper divergence of the southernmost population of the Scots pine (*P. sylvestris nevadensis*, revised by [15]) indicates its more ancient isolation, a common finding for other taxa occurring in Sierra Nevada (e.g. [86, 87]). The clear West–East differentiation of the other Spanish populations was congruent with the mitochondrial and nuclear data from prior literature (Additional file 7). Moreover, the Central Iberian System harbours a boundary between two gene zones (Fig. 4a) resembling the case of *Pinus pinaster* [88] and supporting the role of both sides of this mountain range as refugial areas [18, 19, 57].

Within the “northern” lineage of *G. isabellae*, we have just argued that genetic drift may account for a pre-Holocene origin of the WP cluster (Fig. 1a). This hypothesis would make plausible a Western Pyrenean refuge for this moth, inhabiting that refuge also postulated by Cheddadi et al. [16] and Benito Garzón et al. [18] for *Pinus sylvestris*. These last two works inferred the presence of *P. sylvestris* in the Western Pyrenees during the LGM, although the former presented a much larger potential habitat, covering the Eastern Pyrenees as well. Even assuming an isolated Western Pyrenean LGM refuge for the Scots pine as suggested at Fig. 3 (ECHAM3 scenario) by Benito Garzón et al. [18], we were unable to track the putative forest of *sylvestris/nigra* that connected at some point the Iberian System/Ebro Valley and the Western Pyrenees allowing *G. isabellae* to establish the WP cluster. Maybe the postglacial migration of the so-called “red-haplotype” of *P. sylvestris* from Eastern Iberia to the Western Pyrenees (Fig. 7 by [16]) was somehow singular and left no footprints in the Mid-Holocene expansion scenario displayed in Fig. 4 by [18]. Or maybe that connection occurred before the LGM, as in the case of the plant *Ramonda myconi* [76], and therefore was not reported in any of those works. The LGM-isolation hypothesis of cluster WP receives additional support from the phylogeography of *Carduelis citrinella*, a pine-associated bird, which shows remarkable coincidences with *G. isabellae* in terms of distribution area and genetic structure [81].

We advocate that cluster EP was founded by individuals belonging to EI, independently and after the establishment of cluster WP. The EI-EP corridor was noticeable in the reconstruction of potential habitat for *Pinus nigra* during the LGM and Mid-Holocene [18]. Therefore, we hypothesise that *P. nigra* was the host species that allowed *G. isabellae* to reach the Eastern Pyrenees at some point during the Holocene. It is worth noting that the expansion of *P. sylvestris* from both the Western and Eastern Pyrenean refugia had already covered most of the Spanish slopes of that mountain range at the Mid-Holocene (Fig. 4 by [18]), so the individuals arriving by means of the Black pine found a large area of suitable *P. sylvestris* habitat towards the West. We postulate the Holocene fragmentation of the EI-EP corridor of *P. nigra* as the most likely explanation for (i) an EP locality (Montesquiu) having one specimen carrying the most frequent haplotype (EI.1) of cluster EI, (ii) an EI site (Els-Ports) having four individuals showing the most frequent haplotype (EP.1) of cluster EP, and (iii) the seemingly “misplacement” of haplotype EI.20 being present in only one male from Els-Ports and just one substitution apart from EP.1 (also shared by four individuals from Els-Ports). Testing alternative hypotheses (unidirectional vs. bidirectional geneflow, founder effect vs. gradual fragmentation) for the colonisation of EP will require a more thorough sampling of the Catalonian areas where the distribution of both pine species overlap.

The terminal position of haplotype CI.1 (Fig. 1b) strongly suggests that the Central Iberian System derives from a small number of individuals with Betic ancestry (SI cluster). Indeed, the mismatch distributions of mitochondrial alleles in SI showed unequivocal footprints of a population expansion, likely after the LGM. The foundation of CI could have happened then by means of the northward expansion of *P. sylvestris* from its southernmost glacial refuge, as depicted at Fig. 7 by [16]. Unfortunately, the potential habitat reconstructions for the Scots pine at LGM and mid-Holocene failed to track such a SI-CI connection [18, 19]. The forest connecting SI and CI may have gone unnoticed by the potential habitat reconstructions for *P. sylvestris*, either because of a temporal mismatch or because it actually never existed and *G. isabellae* reached Central Spain by means of its other host-species, *P. nigra.* Both *P. sylvestris* and *P. nigra* were likely to be used by *G. isabellae* when the split between its northern and southern mitochondrial lineages took place before the LGM. Then, the Central Iberian system (CI) was colonised by ancestors of the SI cluster. *P. nigra* was and currently still is most abundant than *P. sylvestris* in South Spain. By contrast, the occurrence of the Black pine in the Central Iberian System is currently minimal. Regardless of the pine species used to arrive to each of these mountain ranges, it is clear that the only possibility for the moth to survive was to use *P. nigra* in the South and *P. sylvestris* in Central Spain (Additional file 7).

### Secondary contacts

We previously discussed that the two reciprocally monophyletic mitochondrial lineages of *G. isabellae* surely remained isolated since the LGM. This explanation is somehow jeopardised by the nuclear results, as the microsatellites revealed the EI cluster to be more closely related to the “southern-lineage”: clusters SI and CI (Figs. 3b, c). According to microsatellites, the SI and EI clusters were connected when populations from EI and the Pyrenees became isolated. This mito-nuclear discordance might be related to male-biased dispersal, a life-history trait reported for *G. isabellae* [89] and invoked to account for the differences in nuclear and mitochondrial differentiation in another pine-dwelling lepidopteran [90]. However, given the geographical scale where the EI mito-nuclear discordance is found, a more plausible explanation is the existence of mito-nuclear incompatibilities, i.e. some kind of post-zygotic mechanism lowering the fitness of those Spanish Moon Moths with “southern lineage” mitochondrial haplotypes when carrying certain “northern” (EI) nuclear genotypes. Given the amount of non-synonymous substitutions in the “southern” lineage it is hardly surprising that selection favoured a specific “southern-mito + southern-nuclear” genetic combination during the period that those populations remained isolated from the rest of conspecifics. After the posterior mating between individuals from SI/CI and EI, the finding of a “southern-mito + northern-nuclear” combination would be expected in our dataset. That absence suggests that selection may have only allowed the “northern-mito + southern-nuclear” combination currently present at the Eastern Iberian System. The differential effect of specific mito-nuclear combinations on the fitness of interpopulation hybrids has been proved in other arthropods such as the copepod *Tigriopus californicus*, *Drosophila (Sophophora) melanogaster* and the seed beetle *Callosobruchus maculatus* (revised by [91, 92]).

A fine-scale survey in the Pyrenees is needed to confirm the presence of *G. isabellae* between Renanué and Baiasca (absent as from [67]) and unveil the factors preventing gene flow between clusters WP and EP. At present, two results from the present work are worth highlighting. The first is the lack of mitochondrial exchange between these two clusters (Fig. 1b). The second is a certain extent of asymmetric nuclear gene flow, as two individuals sampled at Renanué (WP) showed a high EP assignment probability, whereas individuals from Baiasca did not show any WP assignment probability (Fig. 3a). (1) The geographic coincidence of this phylogeographic break and the transition between the two genetically undifferentiated Pyrenean subspecies of *Pinus sylvestris* is remarkable. There are major climatic and ecological differences between the northern and southern slopes of the Pyrenees [93] as well as between the western and eastern parts of this mountain range (e.g. [76, 94–96]). The Spanish Moon Moth obviously needs a suitable host to complete its life cycle, but certain environmental variables, namely precipitation and temperature, have been inferred to be relevant for predicting its current distribution [10]. (2) Our preliminary results ruled out the effect of the maternally inherited bacterium *Wolbachia* as the cause for asymmetric gene flow between the two Pyrenean clusters. The westwards dispersal tracked by the high EP assignment probabilities of two individuals sampled at Renanué (WP) may reflect the onset of their hybridisation mediated by male gene flow. The increase of reforested pine woodland (Additional file 8) reinforces this hypothesis. The geographical pattern of mtDNA variability (higher in the central areas of WP and EP and monomorphic at both ends of each cluster, Fig. 1c) additionally agrees with a leading edge effect. However, further analyses are needed to confirm the aforementioned result as current gene flow.

## Conclusions

*Graellsia isabellae* showed a strong phylogeographic pattern. The six differentiated groups revealed by the microsatellites and mtDNA showed a one to one correspondence with each of the main mountain ranges inhabited by the species. The mitochondrial data further clustered those groups into two major lineages, “southern” and “northern”, which likely diverged before the LGM. There was no evidence of host-associated differentiation between populations using *P. sylvestris* and the ones utilising *P. nigra*. Eastern and Western Pyrenees were most likely colonised by individuals closely related to modern Eastern Iberian populations through independent asynchronous events. The past and present wider distribution of *P. nigra* when compared to *P. sylvestris* suggests that *G. isabellae* used the former to reach several mountain ranges where it currently lives on the latter. This seems to be the case for the populations of the Spanish Moon Moth from the Eastern Pyrenees and French Alps, which likely derived from the Holocene fragmentation of the continuous forest of *P. nigra* connecting different populations of *P. sylvestris* in Northeastern Spain and Southeastern France. The Central Iberian system descends from Betic ancestors, whose populations showed genetic footprints of both a population expansion and posterior bottlenecks. Subsequent gene flow between both the Central/Betic mountains and the Eastern Iberian System was revealed by the nuclear dataset and was compatible with the overlapping potential distribution area of the Scots and Black pines during the Holocene. The mito-nuclear discordance involving the Eastern Iberian cluster is congruent with male-biased gene flow and/or a secondary contact after the evolution of mito-nuclear incompatibilities in geographically isolated areas.

## Abbreviations

Ca, circa; CI, Central Iberia; cpDNA, chloroplast DNA; EI, Eastern Iberia; EP, Eastern Pyrenees; FA, French Alps; HPD, High Posterior Density; ISSR, Inter Simple Sequence Repeat; Kya, kilo (10^3^) years ago; MRCA, most recent common ancestor; mtDNA, mitocondrial DNA; mya, million (10^6^) years ago; na, not applicable; SI, Southern Iberia; SSD, sum of squared deviations; SSR, single sequence repeats; vs, versus; WP, Western Pyrenees.

## Additional files

Additional file 1: Typing for three *Wolbachia* genes. Testing for the presence of *Wolbachia* in 53 specimens of *G. isabellae* following the protocol by Kodandaramaiah et al. [62]. (PDF 1008 kb) Additional file 2: Details of the 95 % Statistical Parsimony network calculated for the 832 bp *COI* fragment of *G. isabellae* (Fig. 1b main text). a) Colours and numbers besides connections indicate the nucleotide where each mutation occurred. Stars point to ambiguous assignations as from software TCS 1.21; b) non-synonymous changes along the alignment. Details of the calculations used to test for no selection (*H*_O:_ d_N_/d_S_ = 1) using MEGA 5; c) discussion. (PDF 180 kb) Additional file 3: Geographic distribution of the mitochondrial haplotypes of *G. isabellae* and accession numbers. (PDF 149 kb) Additional file 4: Genetic variation at microsatellite loci in populations of *G. isabellae*. The two values presented in the “Flobal *F*_IS_ column” correspond to the results of the entire dataset and results excluding loci *GI23* and *GI18*. Significant results at the 5 % after Bonferroni correction are shown in bold. (PDF 34 kb) Additional file 5: Pairwise *F*_ST_ values for 28 localities of *G. isabellae*. Below diagonal: from the mitochondrial dataset (*ϕ*_ST_). Above diagonal: from the microsatellite (nine) loci. Significant values are displayed in bold. Indicative adjusted nominal level (5 %) for multiple comparisons = 0.00013. (PDF 651 kb) Additional file 6; Identification of the most likely number of *G. isabellae* populations by the analysis of microsatellite data with SRUCTURE 2.3.4. a) Estimated log probability of data for the different number of inferred clusters (*K*); bars correspond to standard deviation, after 20 independent runs; b) Rate of change in the log probability of data between successive *K* values (Δk). Both figures were obtained with the aid of STRUCTURE HARVESTER 0.6.94 available at http://taylor0.biology.ucla.edu/struct_harvest/. (PDF 685 kb) Additional file 7: Further details about the phylogeography of *Pinus sylvestris* and P*. nigra* and their relationship with *G. isabellae*. Population structure of *P. sylvestris* and *P. nigra* as revealed by different molecular markers. All figures are reproduced with kind permission of the copyright holder. (PDF 1273 kb) Additional file 8: Detailed distribution of *Pinus sylvestris* in the Spanish Pyrenees, both natural (green) and reforested (blue) areas. a) Municipalities where samples of *G. isabellae* were obtained are highlighted; b) Enlarged view of the area between Baiasca (*L9*) and Renanué (*L10*). (PDF 3010 kb)
