# Supplementary material for: Phylogeography of the Spanish Moon Moth Graellsia isabellae (Lepidoptera, Saturniidae)
Source: BMC Evol Biol. 2016 Jun 24;16:139. doi: 10.1186/s12862-016-0708-y (PMC4919910; doi:10.1186/s12862-016-0708-y)
Supplement: Additional file 1: — Typing for three Wolbachia genes. Testing for the presence of Wolbachia in 53 specimens of G. isabellae following the protocol by Kodandaramaiah et al. [62]. (PDF 1008 kb) [file 12862_2016_708_MOESM1_ESM.pdf]

Additional file 1: Typing for three *Wolbachia* genes

The mitochondrial and nuclear discordant levels of differentiation between the Pyrenean sites *L9* and *L10* might have been caused by reproductive parasites such as *Wolbachia*. We tested for presence of this bacteria in a set of 53 specimens of *Graellsia* following a standard protocol [1].

We discarded marker *wsp* after the pilot testing, as it amplified in *Drosophila* spp., but not in the Lepidopteran positive controls (**Figure I**).

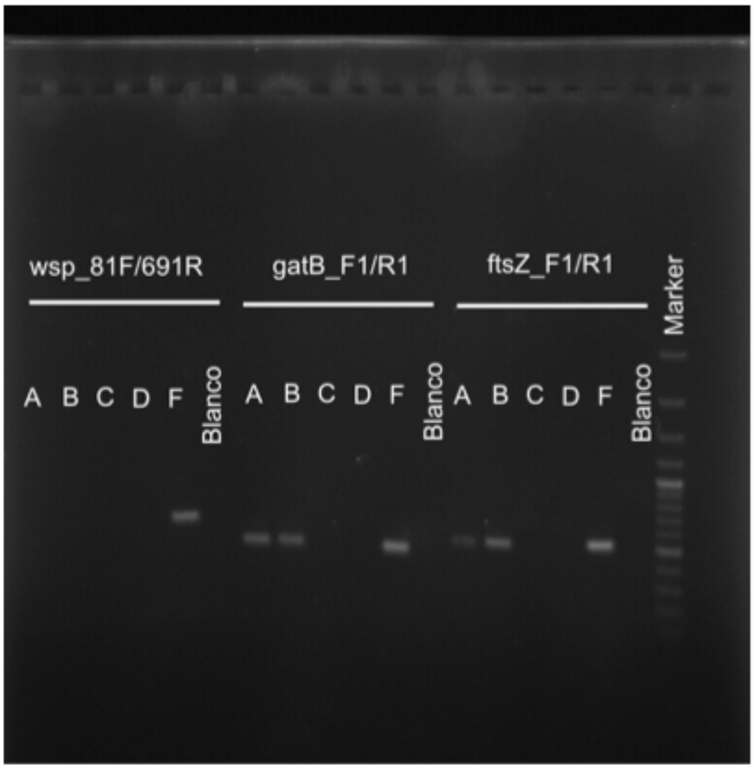

Figure I: Positive (tubes A and B) and negative (tubes C and D) controls for *Wolbachia* infection on Lepidoptera. An extra positive control was also assayed (tube F, *Drosophila* spp.). "Blanco" (*blank*) stands for PCR negative control.

Markers *gatB* and *ftsZ* were amplified in 53 samples of *Graellsia isabellae*: ten from Eastern Pyrenees (one from Baiasca, *L9*, and nine from its immediate vicinity), ten from the Western Pyrenees (Renanué, *L10*), ten from Southern Iberia (Cazorla, *L27*),

five from Central Iberia (Cercedilla, *L22*), five from the French Alps (Ange-Gardien, *L16*) and 13 from Eastern Iberia (ten from Els-Ports, *L4*, and three more from the Eastern Iberian System, i.e. Ademuz (*L1*), Bronchales (*L2*) and a third locality called Fuente Cabrito 40º10'13''N 1º3'51''W). It is worth mentioning that the three WP individuals from Renanué (*L10*) (REN8, RN16 and REN19) with high membership to EP as from STRUCTURE (Figure 2 main text) were part of this testing. The same applies to individual CAZ16, sampled at Cazorla (*L27*) but with a high affinity with the Eastern Iberian cluster.

PCRs were carried out in a final volume of 25 µL, containing 6.50 µL of Supreme NZYTaq Green PCR Master Mix (NZYTech), 0.5 µM of each primer, 25 ng of template DNA, extracted from leg tissue, and PCR-grade water up to 25 µL. The thermal cycling conditions were as follows: an initial denaturation step at 95 ºC for 5 min, followed by 35 cycles of denaturation at 95 ºC for 30 s; annealing at 55 ºC for both primer pairs; extension at 72 ºC for 45 s; and a final extension step at 72 ºC for 5 min.

A negative control that contained no DNA was included in every PCR round to check for cross-contamination during the experiments. Likewise, a positive control, consisting on a DNA sample from a *Wolbachia*-infected butterfly individual, was included to ensure that the reactions worked well. PCR products were run on 1 % agarose gels stained with Real Safe (Durviz), and imaged under UV light.

The agarose gel images (**Figure II**) showed no amplification of any DNA fragment of the expected size. PCRs made with the *gatB* primers showed unspecific amplification of several bands of around 300, 700, and 1000 bp, but no amplification at the expected 500 bp size. PCRs made with the *ftsZ* primers showed no amplification at all. Both *gatB* and *ftsZ* positive control samples yielded a sharp band at around 500 bp.

None of the negative controls showed any amplification, as expected.

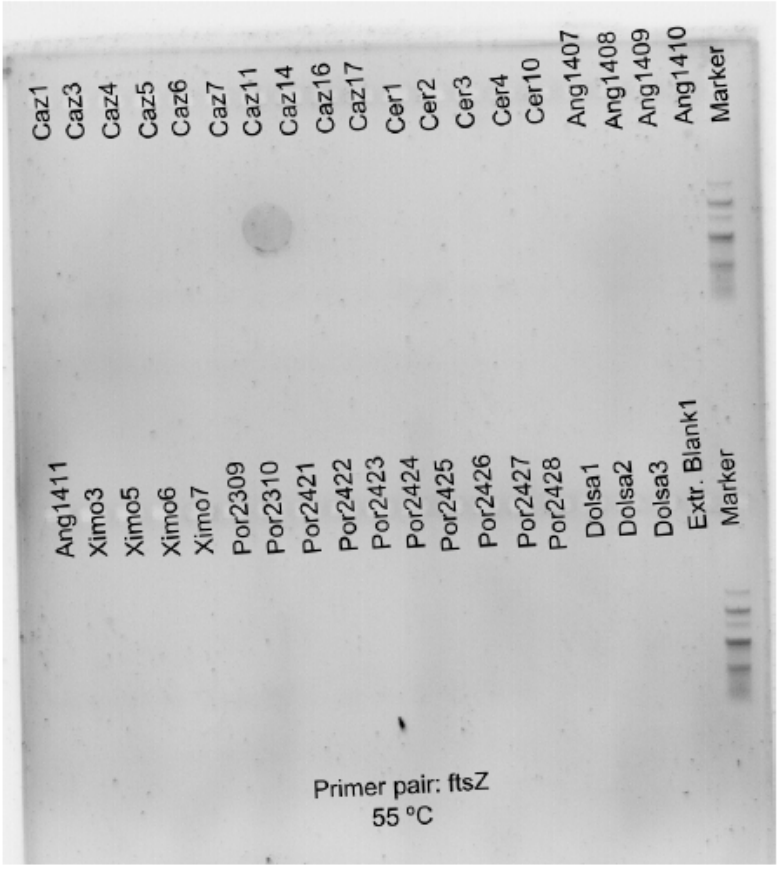

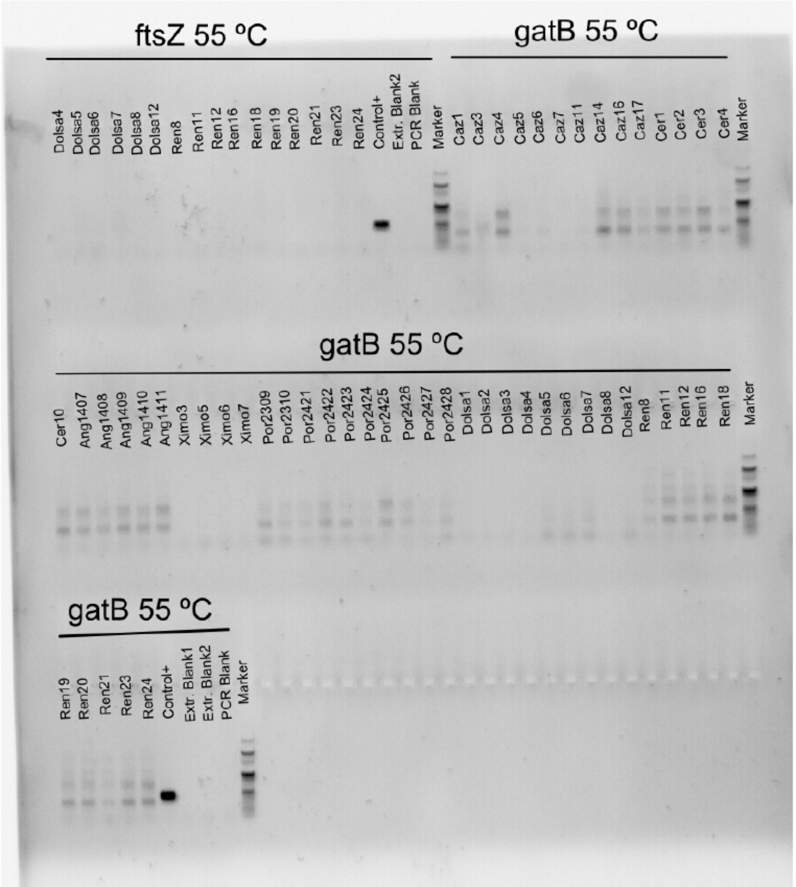

Figure II: PCR gels under UV light.

Reference:

1. Kodandaramaiah U, Simonsen TJ, Bromilow S, Wahlberg N, Sperling FAH. Deceptive single-locus taxonomy and phylogeography: *Wolbachia* associated divergence in mitochondria DNA is not reflected in morphology and nuclear markers in a butterfly species. Ecol. Evol. 2013; 3:5167-5176.
