# Supplementary material for: Phylogeography of the Spanish Moon Moth Graellsia isabellae (Lepidoptera, Saturniidae)
Source: BMC Evol Biol. 2016 Jun 24;16:139. doi: 10.1186/s12862-016-0708-y (PMC4919910; doi:10.1186/s12862-016-0708-y)
Supplement: Additional file 2: — Details of the 95 % Statistical Parsimony network calculated for the 832 bp COI fragment of G. isabellae (Fig. 1b main text). a) Colours and numbers besides connections indicate the nucleotide where each mutation occurred. Stars point to ambiguous assignations as from software TCS 1.21; b) non-synonymous changes along the alignment. Details of the calculations used to test for no selection (H O: dN/dS = 1) using MEGA 5; c) discussion. (PDF 180 kb) [file 12862_2016_708_MOESM2_ESM.pdf]

Additional file 2. Details of the 95% Statistical Parsimony network calculated for the 832 bp *COI* fragment from *G. isabellae* (Figure 1b main text).

a) Colours and numbers besides connections indicate the nucleotide where each mutation occurred. Stars point to ambiguous assignations as from software TCS 1.21.

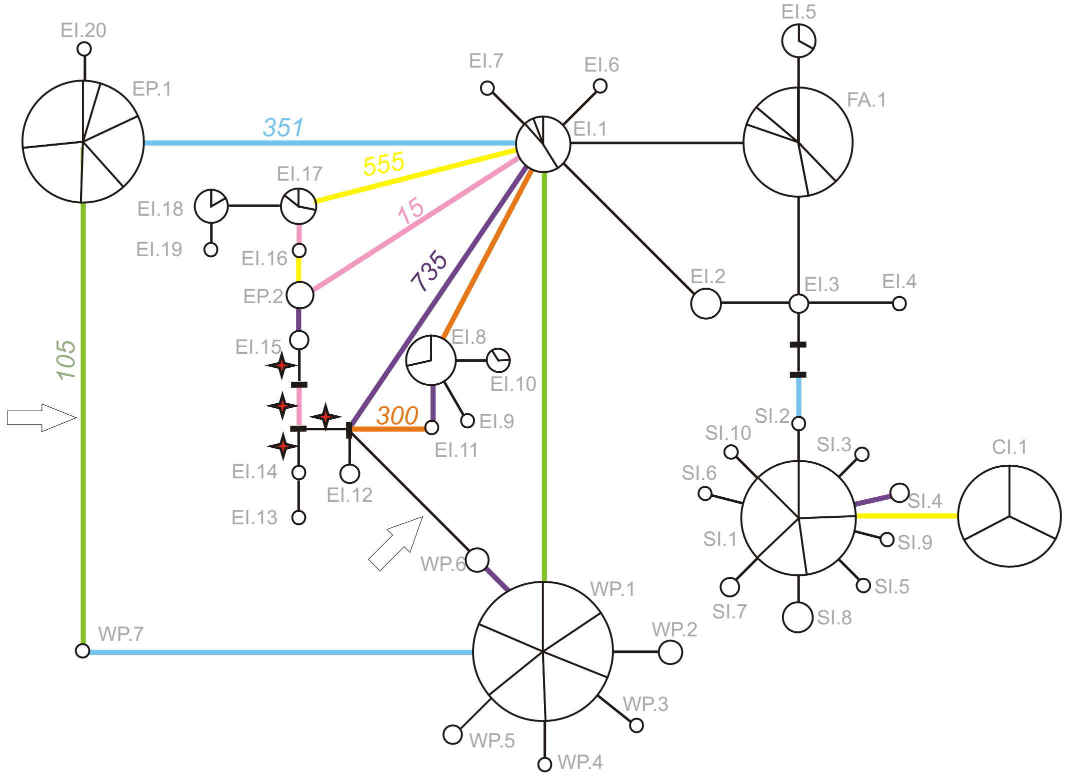

b) Non-synonymous changes along the alignment. We calculated the dN/dS ratio (1000 bootstrap replications were used to estimate variances) and then tested the null hypothesis of no selection (H0: dN=dS) versus the purifying selection hypothesis (H1: dN<dS) using a one-tailed codon based Z-test: $Z = (dN-dS)/\sqrt{(Var(dS)+Var(dN))}$ and the “Kumar method (Kimura 2-para)” as substitution model. All these calculations were performed using MEGA5 [1].

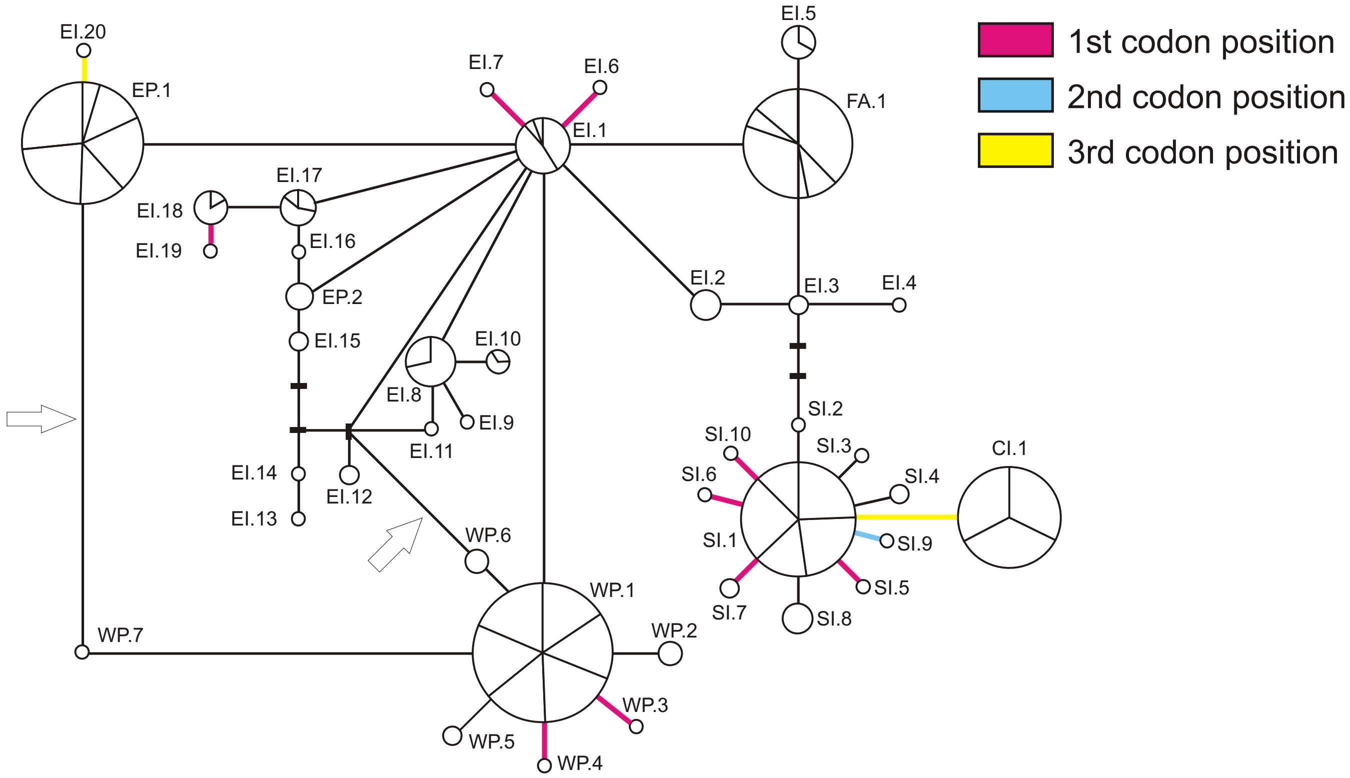

c) Discussion.

There were twelve non-synonymous substitutions (out of 34) scattered all along the mitochondrial *COI* sequences obtained from *G. isabellae*. All of them occurred at terminal haplotypes, particularly as part of the "southern" lineage (clusters SI-CI). The ratio of within-lineages non-synonymous and synonymous nucleotide polymorphisms of the *COI* gene pointed to purifying selection to be acting on clusters EI, WP, EP and FA, whereas the null-hypothesis $d_N/d_S = 1$ could not be rejected for the SI-CI group. This result should be taken with caution, as the use of d_N/d_S ratios to test for selection at intraspecific level is controversial [2]. In addition, the no rejection of the null hypothesis is usually interpreted as due to largely neutral amino-acid substitutions (in this case on the southern lineage). However, such a result may be also due to positive selection cancelling purifying selection. Mutation, selection, demography and even statistical artefacts may have led to the observed non-synonymous substitutions (revised by [2]). Surveying the process causing the accumulation of amino-acid changes on one of the lineages of *G. isabellae* is beyond the scope of this work. However, it is worth noting that the SI cluster, using *P. nigra* and perhaps *P. halepensis/P. pinaster* as host-species, showed evidence of population fluctuations (Tables 1 and 2, Figures 1b and 2b). Therefore, plausible hypotheses accounting for the prevalence of non-synonymous changes on the SI cluster would be (i) positive selection (revised by [3]), caused by the hypothetically different southern environmental conditions; (ii) the release of strong evolutionary constraints during a population expansion (e.g. [4]), a result reported for the star-like SI cluster and (iii) the fact that non-synonymous changes are expected to occur more frequently in small populations, as slightly deleterious variants may be fixed by genetic drift then [5]. The population-size effect on the fixation of deleterious mutations is more severe in genes under strong purifying selection [6], which was

proved to act on the mitochondrial *COI* gene and conditioned the evolution of three divergent haplogroups of *Drosophila simulans* [7]. Although inconclusive, our results prepare the ground for future research confirming purifying selection on the *COI* gene and testing whether the northern lineage maintained a more stable environment keeping the populations of *G. isabellae* large enough to remove slightly deleterious mutations, whereas the demographic and/or environmental changes suffered by the southern lineage led to the fixation of such "disadvantageous" variants (e.g. [8]).

References

1. Tamura K, Peterson D, Peterson N, Stecher G, Nei M, Kumar S. MEGA5: Molecular Evolutionary Genetics Analysis using Maximum Likelihood, Evolutionary Distance, and Maximum Parsimony Methods. Mol Biol Evol. 2011;28: 2731-9.
2. Kryazhimskiy S, Plotkin J. The Population Genetics of dN/dS. PloS Genet. 2008;4.
3. Castellana S, Vicario S, Saccone C. Evolutionary Patterns of the Mitochondrial Genome in Metazoa: Exploring the Role of Mutation and Selection in Mitochondrial Protein-Coding Genes. Genome Biol Evol. 2011;3:1067-79.
4. Zsurka G, Kudina T, Peeva V, Hallmann K, Elger C, Khrapko K, et al. Distinct patterns of mitochondrial genome diversity in bonobos (*Pan paniscus*) and humans. BMC Evol Biol. 2010;10.
5. Ohta T. Amino acid substitution at the *Adh* locus of *Drosophila* is facilitated by small population size. Proc Natl Acad Sci U S A. 1993;90:4548-51.
6. Subramanian S. Significance of Population Size on the Fixation of Nonsynonymous Mutations in Genes Under Varying Levels of Selection Pressure. Genetics. 2013;193:995-1002.

7. Dean M, Ballard J. High divergence among *Drosophila simulans* mitochondrial haplogroups arose in midst of long term purifying selection. Mol Phylogenet Evol. 2005;36:328-37.
8. Shirai K, Inomata N, Mizoiri S, Aibara M, Terai Y, Okada N, et al. High prevalence of non-synonymous substitutions in mtDNA of cichlid fishes from Lake Victoria. Gene. 2014;552:239-45.
