# Supplementary material for: Phylogeography of the Spanish Moon Moth Graellsia isabellae (Lepidoptera, Saturniidae)
Source: BMC Evol Biol. 2016 Jun 24;16:139. doi: 10.1186/s12862-016-0708-y (PMC4919910; doi:10.1186/s12862-016-0708-y)
Supplement: Additional file 3: — Geographic distribution of the mitochondrial haplotypes of G. isabellae and accession numbers. (PDF 149 kb) [file 12862_2016_708_MOESM3_ESM.pdf]

Additional file 3. Geographic distribution of the mitochondrial haplotypes of *Graellsia isabellae* and accession numbers.

	Population	Ademuz	Bronchales	Huerta	Els Ports	Albanyà	Castellfolit	Montesquiu	Montgrony	Baiasca	Renanué	Sarra	Ordesa	Cabas	S. Juan de la Peña	Belabarce	Ange Gardien	Fournel	Cristillan	Guillestre	Auzet	Rascafría	Cercedilla	Peguerinos	Río Mundo	Guillimona	Sagra	Cazorla	Sierra María
Genebank	Population Code	*L1*	*L2*	*L3*	*L4*	*L5*	*L6*	*L7*	*L8*	*L9*	*L10*	*L11*	*L12*	*L13*	*L14*	*L15*	*L16*	*L17*	*L18*	*L19*	*L20*	*L21*	*L22*	*L23*	*L24*	*L25*	*L26*	*L27*	*L28*
Accession #	Haplotype Code																												
KX302370	FA.1																25	22	4	9	6								
KX302371	WP.1										20	19	17	17	16	20													
KX302389	WP.2														3														
KX302379	WP.3													1															
KX302381	WP.4													1															
KX302390	WP.5														2														
KX302396	WP.6												3																
KX302380	WP.7													1															
KX302369	EP.1				4	11	17	19	10	22																			
KX302393	EP.2								4																				
KX302361	EI.1	7		1	8			1																					
KX302397	EI.2				5																								
KX302367	EI.3	2																											
KX302365	EI.4	1																											
KX302373	EI.5		2	4																									
KX302375	EI.6		1																										
KX302398	EI.7				1																								
KX302374	EI.8		10	4																									
KX302376	EI.9		1																										
KX302377	EI.10		1	2																									
KX302387	EI.11			1																									
KX302363	EI.12	2																											
KX302364	EI.13	1																											
KX302362	EI.14	1																											
KX302388	EI.15			2																									
KX302368	EI.16	1																											
KX302366	EI.17	2	4	1																									
KX302378	EI.18		1	5																									
KX302372	EI.19		1																										
KX302399	EI.20				1																								
KX302382	SI.1																								17	11	18	9	18
KX302394	SI.2																								1				
KX302401	SI.3																										1		
KX302386	SI.4																									2			
KX302391	SI.5																												1
KX302392	SI.6																												1
KX302384	SI.7																											2	
KX302383	SI.8																											5	
KX302400	SI.9																										1		
KX302395	SI.10																								1				
KX302385	CI.1																					19	19	20					
