# Supplementary material for: Phylogeography of the Spanish Moon Moth Graellsia isabellae (Lepidoptera, Saturniidae)
Source: BMC Evol Biol. 2016 Jun 24;16:139. doi: 10.1186/s12862-016-0708-y (PMC4919910; doi:10.1186/s12862-016-0708-y)
Supplement: Additional file 4: — Genetic variation at microsatellite loci in populations of G. isabellae. The two values presented in the “Flobal F IS column” correspond to the results of the entire dataset and results excluding loci GI23 and GI18. Significant results at the 5 % after Bonferroni correction are shown in bold. (PDF 34 kb) [file 12862_2016_708_MOESM4_ESM.pdf]

Additional file 4. Genetic variation at microsatellite loci in populations of *G.isabellae*. The two values presented in the "Global F_{IS} column" correspond to the results of the entire dataset and results excluding loci *GI23* and *GI18*. Significant results at the 5% after Bonferroni correction are shown in bold.

	All loci			F_{IS}, NA and H_{WE} for each locus and population								
Population	He	H_{WE} (*p*-value)	Global F_{IS}	GI23	GI6	GI17	GI11	GI15	GI21	GI25	GI18	GI26
			**0.104**	0.014	0.206	0.174	0.04	0.049	-0.034	-0.094	**0.569**	-0.088
Ademuz (*L1*)	0.731	**0.0000**	0.054	6	12	7	21	6	12	9	6	5
				0.761	0.905	0.795	0.944	0.69	0.786	0.743	0.696	0.259
			**0.106**	0.084	0.032	0.063	0.143	0.021	-0.008	0.114	**0.422**	0.07
Bronchales (*L2*)	0.755	0.0025	-0.042	6	17	8	14	6	10	10	8	6
				0.74	0.929	0.791	0.828	0.725	0.827	0.728	0.807	0.416
			**0.116**	0.243	0.13	0.096	-0.014	-0.004	0.089	0.101	0.402	-0.088
H.del Marque.(*L3*)	0.760	**0.0001**	0.013	6	16	7	22	7	15	8	7	5
				0.743	0.89	0.83	0.924	0.685	0.85	0.73	0.784	0.402
			0.061	-0.058	0.043	0.037	0.008	-0.129	0.083	0.032	**0.566**	-0.165
Els Ports (*L4*)	0.749	0.0103	-0.413	7	17	7	17	6	16	7	8	4
				0.761	0.911	0.81	0.907	0.775	0.682	0.705	0.81	0.376
			0.075	1.000	0.016	-0.176	-0.183	-0.127	-0.071	-0.071	-0.119	0.195
Albanyà (*L5*)	0.481	0.3727	-0.061	2	3	2	5	3	3	3	3	4
				0.533	0.555	0.309	0.768	0.323	0.255	0.255	0.65	0.677
			0.207	**1.000**	-0.199	0	-0.053	-0.113	-0.086	0.325	0.2	**0.634**
Castellfolit (*L6*)	0.475	**0.0005**	0.095	2	2	2	5	3	3	3	3	4
				0.425	0.51	0.056	0.739	0.449	0.511	0.412	0.417	0.758
			**0.123**	**0.895**	-0.023	0.304	0.053	0.142	0.067	-0.131	0.152	0.098
Montequiu (*L7*)	0.493	**0.0000**	0.061	3	5	2	8	4	4	4	3	4
				0.34	0.6	0.101	0.722	0.348	0.489	0.481	0.662	0.693
			**0.139**	**0.636**	-0.027	0.244	0.095	-0.029	-0.226	0.012	0.215	0.362
Montgrony (*L8*)	0.521	**0.0002**	0.039	3	5	3	8	3	3	4	3	4
				0.493	0.584	0.147	0.762	0.389	0.526	0.517	0.623	0.649
			-0.061	NA	0.068	-0.067	-0.064	-0.185	-0.026	-0.307	-0.054	0.062
Baiasca (*L9*)	0.460	0.7509	0.110	1	4	2	6	3	4	3	5	4
				0	0.429	0.15	0.789	0.473	0.663	0.428	0.495	0.711
			0.074	1.000	0.182	0.269	-0.091	-0.072	-0.09	-0.039	0.15	0.098
Renanué (*L10*)	0.544	0.0227	0.041	2	5	4	8	4	3	3	4	4
				0.077	0.693	0.662	0.798	0.662	0.592	0.124	0.645	0.644
			-0.000	1.000	-0.17	0.184	-0.024	0.098	-0.27	-0.054	-0.036	0.054
La Sarra (*L11*)	0.560	0.3295	0.035	2	5	5	7	5	3	3	4	3
				0.091	0.738	0.746	0.799	0.723	0.573	0.165	0.629	0.577
			0.112	NA	-0.213	0.477	0.017	0.059	0.441	0.268	0.099	-0.054
Ordesa (*L12*)	0.542	0.0159	0.047	1	5	4	7	4	4	2	4	3
				0	0.702	0.566	0.754	0.709	0.663	0.202	0.617	0.667
			0.066	0.488	-0.067	0.407	-0.016	0.014	0.072	NA	0.151	-0.071
Cabas (*L13*)	0.547	0.0131	0.151	3	5	5	9	4	6	1	4	3
				0.122	0.732	0.527	0.83	0.698	0.64	0	0.699	0.671
			0.149	NA	0.071	0.411	-0.038	-0.206	0.047	0.35	0.476	0.216
S.Juan P. (*L14*)	0.551	**0.0016**	0.415	1	8	5	10	3	4	3	3	3
				0	0.717	0.707	0.883	0.518	0.656	0.321	0.556	0.638
			0.102	1.000	0.257	0.444	0.008	0.034	-0.083	0.529	0.049	-0.26
Belabarce (*L15*)	0.511	**0.0010**	-0.038	2	5	3	6	3	3	2	3	3
				0.065	0.652	0.62	0.781	0.568	0.625	0.205	0.526	0.558
			0.054	1.000	NA	-0.026	0.119	NA	-0.068	-0.114	NA	0.009
Ange Gardien (*L16*)	0.135	0.0189	0.055	2	1	2	4	1	2	2	1	2
				0.05	0	0.073	0.397	0	0.14	0.202	0	0.353
			0.151	NA	NA	0.045	0.056	NA	0.326	-0.077	NA	0.645
Fournel (*L17*)	0.156	0.0063	0.109	1	1	2	2	1	3	2	1	2
				0	0	0.407	0.441	0	0.247	0.155	0	0.156
			0.047	NA	NA	NA	0.431	NA	0	-0.1	NA	-0.294
Cristillan (*L18*)	0.126	0.4893	0.052	1	1	1	2	1	2	2	1	2
				0	0	0	0.439	0	0.083	0.227	0	0.386
			0.415	NA	0	NA	0.552	NA	-0.176	-0.053	NA	0.828
Guillestre (*L19*)	0.190	0.0066	0.057	1	2	1	3	1	2	2	1	2
				0	0.091	0	0.609	0	0.309	0.173	0	0.527
			-0.413	NA	NA	-0.429	0	NA	-0.25	-0.667	NA	NA
Auzet (*L20*)	0.170	1.0000	0.041	1	1	2	2	1	2	2	1	1
				0	0	0.467	0.167	0	0.4	0.5	0	0
			0.114	NA	0.088	0.151	0.239	NA	-0.043	0.238	0	0.114
Rascafría (*L21*)	0.398	0.0749	0.030	1	4	2	4	1	4	2	2	2
				0	0.62	0.512	0.628	0	0.667	0.514	0.348	0.294
			0.077	NA	-0.13	-0.445	0.469	NA	0.071	0.208	0.508	-0.132
Cercedilla (*L22*)	0.390	0.0911	0.114	1	4	2	5	1	4	2	5	3
				0	0.46	0.498	0.678	0	0.603	0.505	0.325	0.53
			**0.209**	NA	0.132	0.36	0.356	NA	-0.03	0.237	0.379	0.053
Peguerinos (*L23*)	0.386	0.0089	0.187	1	3	3	4	1	4	2	4	3
				0	0.504	0.488	0.628	0	0.546	0.41	0.403	0.494
			0.166	0.174	-0.08	0.025	0.03	-0.033	0	0.112	**0.882**	0.468
Río Mundo (*L24*)	0.581	**0.0000**	-0.001	2	13	3	8	3	7	5	6	2
				0.385	0.884	0.513	0.843	0.132	0.818	0.716	0.768	0.171
			0.031	-0.235	0.026	-0.016	0.077	-0.043	-0.184	-0.178	0.518	0
Guilimona (*L25*)	0.573	0.1600	0.126	3	7	4	11	4	6	3	5	2
				0.436	0.869	0.606	0.917	0.221	0.715	0.522	0.798	0.077
			0.080	-0.008	-0.019	-0.179	0.009	-0.036	0.011	0.351	**0.453**	-0.015
Sagra (*L26*)	0.592	0.0637	0.045	3	11	5	13	5	14	5	4	2
				0.312	0.841	0.654	0.894	0.331	0.862	0.66	0.72	0.056
			0.086	0.166	-0.012	0.159	0.012	0.061	-0.016	0.103	0.295	0
Cazorla (*L27*)	0.592	0.0188	0.024	3	11	4	13	4	10	4	7	2
				0.473	0.865	0.648	0.893	0.219	0.839	0.656	0.709	0.03
			0.105	0.362	0.154	-0.056	-0.041	0.089	-0.037	0.248	0.329	-0.14
María (*L28*)	0.484	**0.0015**	-0.016	2	4	3	8	2	6	5	5	3
				0.152	0.591	0.153	0.775	0.496	0.653	0.601	0.673	0.263
