# Supplementary material for: Phylogeography of the Spanish Moon Moth Graellsia isabellae (Lepidoptera, Saturniidae)
Source: BMC Evol Biol. 2016 Jun 24;16:139. doi: 10.1186/s12862-016-0708-y (PMC4919910; doi:10.1186/s12862-016-0708-y)
Supplement: Additional file 5: — Pairwise F ST values for 28 localities of G. isabellae. Below diagonal: from the mitochondrial dataset (ϕ ST). Above diagonal: from the microsatellite (nine) loci. Significant values are displayed in bold. Indicative adjusted nominal level (5 %) for multiple comparisons = 0.00013. (PDF 651 kb) [file 12862_2016_708_MOESM5_ESM.pdf]

Additional file 5. Pairwise F_{ST} values for 28 localities of *Graellsia isabellae*. Below diagonal: from the mitochondrial dataset (Φ_{ST}).

Above diagonal: from the microsatellite (nine) loci.

Significant values are displayed in bold. Indicative adjusted nominal level (5%) for multiple comparisons = 0.00013.

	EI				EP					WP						FA					CI			SI				
	ADE *L1*	BRO *L2*	HUE *L3*	POR *L4*	ALB *L5*	CAS *L6*	MTQ *L7*	MOG *L8*	BAI *L9*	REN *L10*	SAR *L11*	ORD *L12*	CAB *L13*	JUA *L14*	BEL *L15*	ANG *L16*	FOU *L17*	CRI *L18*	GUIL *L19*	AUZ *L20*	RAS *L21*	CER *L22*	PEG *L23*	MUN *L24*	GUI *L25*	SAG *L26*	CAZ *L27*	MAR *L28*
ADE *L1*	*	0.011	0.008	**0.030**	**0.262**	**0.253**	**0.256**	**0.221**	**0.240**	**0.174**	**0.162**	**0.195**	**0.120**	**0.191**	**0.197**	**0.470**	**0.450**	**0.379**	**0.337**	**0.339**	**0.244**	**0.244**	**0.261**	**0.117**	**0.098**	**0.111**	**0.120**	**0.179**
BRO *L2*	0.139	*	0.010	**0.020**	**0.245**	**0.238**	**0.242**	**0.214**	**0.231**	**0.151**	**0.146**	**0.173**	**0.180**	**0.173**	**0.186**	**0.476**	**0.452**	**0.384**	**0.347**	**0.330**	**0.218**	**0.232**	**0.253**	**0.117**	**0.113**	**0.119**	**0.120**	**0.198**
HUE *L3*	0.061	0.012	*	0.027	**0.225**	**0.224**	**0.231**	**0.198**	**0.216**	**0.157**	**0.136**	**0.172**	**0.171**	**0.158**	**0.175**	**0.436**	**0.416**	**0.347**	**0.306**	**0.311**	**0.219**	**0.216**	**0.229**	**0.114**	**0.098**	**0.105**	**0.111**	**0.185**
POR *L4*	0.089	**0.260**	**0.194**	*	**0.228**	**0.223**	**0.228**	**0.197**	**0.219**	**0.137**	**0.150**	**0.170**	**0.175**	**0.160**	**0.171**	**0.452**	**0.433**	**0.374**	**0.338**	**0.343**	**0.252**	**0.259**	**0.276**	**0.165**	**0.147**	**0.151**	**0.161**	**0.226**
ALB *L5*	**0.433**	**0.522**	**0.425**	0.463	*	0.037	0.058	0.048	**0.145**	**0.233**	**0.222**	**0.239**	**0.205**	**0.198**	**0.257**	**0.350**	**0.342**	**0.268**	0.238	0.375	**0.444**	**0.465**	**0.475**	**0.406**	**0.422**	**0.389**	**0.401**	**0.452**
CAS *L6*	**0.494**	**0.573**	**0.480**	**0.519**	0.000	*	0.020	0.008	**0.126**	**0.438**	**0.227**	**0.229**	**0.214**	**0.177**	**0.245**	**0.401**	**0.405**	**0.314**	**0.275**	**0.399**	**0.418**	**0.472**	**0.483**	**0.390**	**0.405**	**0.405**	**0.380**	**0.428**
MTQ *L7*	**0.483**	**0.563**	**0.474**	**0.484**	-0.033	-0.008	*	0.019	**0.090**	**0.223**	**0.220**	**0.238**	**0.217**	**0.188**	**0.244**	**0.266**	**0.284**	**0.216**	**0.190**	**0.328**	**0.435**	**0.455**	**0.464**	**0.381**	**0.395**	**0.361**	**0.378**	**0.413**
MOG *L8*	**0.279**	**0.391**	**0.298**	0.237	0.198	0.259	0.212	*	**0.083**	**0.209**	**0.214**	**0.229**	**0.214**	**0.179**	**0.234**	**0.323**	**0.338**	**0.261**	**0.226**	**0.359**	**0.383**	**0.424**	**0.432**	**0.356**	**0.367**	**0.338**	**0.349**	**0.394**
BAI *L9*	**0.533**	**0.606**	**0.516**	**0.556**	0.000	0.000	0.005	0.299	*	**0.212**	**0.237**	**0.231**	**0.218**	**0.194**	**0.269**	**0.415**	**0.419**	**0.334**	**0.286**	**0.411**	**0.476**	**0.500**	**0.501**	**0.360**	**0.379**	**0.344**	**0.363**	**0.409**
REN *L10*	**0.518**	**0.594**	**0.502**	**0.691**	**1.000**	**1.000**	**0.974**	**0.815**	**1.000**	*	**0.060**	**0.094**	**0.104**	**0.102**	**0.116**	**0.486**	**0.460**	**0.405**	**0.364**	**0.405**	**0.393**	**0.424**	**0.434**	**0.316**	**0.307**	**0.291**	**0.303**	**0.369**
SAR *L11*	**0.510**	**0.587**	**0.495**	**0.685**	**1.000**	**1.000**	**0.974**	**0.810**	**1.000**	0.000	*	**0.087**	**0.054**	**0.089**	**0.047**	**0.443**	**0.405**	**0.354**	**0.307**	**0.367**	**0.416**	**0.412**	**0.424**	**0.295**	**0.284**	**0.264**	**0.280**	**0.356**
ORD *L12*	**0.474**	**0.565**	**0.473**	**0.640**	**0.920**	**0.933**	**0.912**	**0.754**	**0.941**	0.105	0.101	*	0.020	**0.055**	**0.093**	**0.471**	**0.430**	**0.388**	**0.341**	**0.369**	**0.436**	**0.454**	**0.465**	**0.294**	**0.303**	**0.297**	**0.301**	**0.371**
CAB *L13*	**0.482**	**0.559**	**0.476**	**0.625**	**0.906**	**0.921**	**0.900**	**0.736**	**0.930**	-0.000	-0.003	0.053	*	**0.047**	**0.063**	**0.405**	**0.358**	**0.328**	**0.287**	**0.334**	**0.436**	**0.436**	**0.446**	**0.312**	**0.317**	**0.311**	**0.317**	**0.382**
JUA *L14*	**0.477**	**0.552**	**0.473**	**0.613**	**0.873**	**0.892**	**0.875**	**0.722**	**0.904**	0.076	0.072	0.089	0.048	*	**0.058**	**0.449**	**0.418**	**0.359**	**0.313**	**0.369**	**0.423**	**0.454**	**0.467**	**0.297**	**0.309**	**0.288**	**0.294**	**0.349**
BEL *L15*	**0.518**	**0.594**	**0.502**	**0.691**	**1.000**	**1.000**	**0.974**	**0.815**	**1.000**	0.000	0.000	0.105	-0.000	0.076	*	**0.467**	**0.498**	**0.390**	**0.347**	**0.401**	**0.449**	**0.457**	**0.466**	**0.327**	**0.322**	**0.297**	**0.307**	**0.380**
ANG *L16*	**0.463**	**0.589**	**0.448**	**0.719**	**1.000**	**1.000**	**0.977**	**0.836**	**1.000**	**1.000**	**1.000**	**0.944**	**0.938**	**0.910**	**1.000**	*	0.054	-0.021	0.027	**0.463**	**0.704**	**0.679**	**0.652**	**0.634**	**0.674**	**0.576**	**0.600**	**0.644**
FOU *L17*	**0.442**	**0.571**	**0.428**	**0.702**	**1.000**	**1.000**	**0.976**	**0.824**	**1.000**	**1.000**	**1.000**	**0.941**	**0.934**	**0.904**	**1.000**	0.000	*	0.055	0.103	**0.352**	**0.688**	**0.661**	**0.637**	**0.609**	**0.649**	**0.558**	**0.579**	**0.626**
CRI *L18*	0.218	0.384	0.224	**0.534**	1.000	**1.000**	**0.956**	0.674	**1.000**	**1.000**	**1.000**	0.896	**0.884**	**0.838**	**1.000**	0.000	0.000	*	0.009	**0.480**	**0.641**	**0.616**	**0.604**	**0.543**	**0.579**	**0.496**	**0.521**	**0.572**
GUIL *L19*	**0.316**	**0.463**	**0.314**	**0.604**	**1.000**	**1.000**	**0.964**	**0.739**	**1.000**	**1.000**	**1.000**	**0.914**	**0.904**	**0.865**	**1.000**	0.000	0.000	0.000	*	0.412	**0.608**	**0.583**	**0.576**	**0.501**	**0.526**	**0.459**	**0.484**	**0.536**
AUZ *L20*	0.268	0.423	0.270	**0.567**	**1.000**	**1.000**	**0.960**	**0.705**	**1.000**	**1.000**	**1.000**	**0.904**	**0.893**	**0.850**	**1.000**	0.000	0.000	0.000	0.000	*	**0.602**	**0.576**	**0.587**	**0.474**	**0.511**	**0.460**	**0.472**	**0.535**
RAS *L21*	**0.851**	**0.876**	**0.830**	**0.922**	**1.000**	**1.000**	**0.992**	**0.943**	**1.000**	**1.000**	**1.000**	**0.983**	**0.981**	**0.972**	**1.000**	**1.000**	**1.000**	1.000	**1.000**	**1.000**	*	**0.187**	**0.200**	**0.335**	**0.358**	**0.328**	**0.326**	**0.433**
CER *L22*	**0.851**	**0.876**	**0.830**	**0.922**	**1.000**	**1.000**	**0.992**	**0.943**	**1.000**	**1.000**	**1.000**	**0.983**	**0.981**	**0.972**	**1.000**	**1.000**	**1.000**	**1.000**	**1.000**	**1.000**	0.000	*	0.013	**0.333**	**0.343**	**0.323**	**0.337**	**0.455**
PEG *L23*	**0.855**	**0.879**	**0.834**	**0.924**	**1.000**	**1.000**	**0.992**	**0.945**	**1.000**	**1.000**	**1.000**	**0.984**	**0.981**	**0.973**	**1.000**	**1.000**	**1.000**	1.000	**1.000**	**1.000**	0.000	0.000	*	**0.342**	**0.355**	**0.333**	**0.348**	**0.456**
MUN *L24*	**0.816**	**0.849**	**0.800**	**0.889**	**0.973**	**0.978**	**0.970**	**0.911**	**0.981**	**0.985**	**0.985**	**0.984**	**0.964**	**0.954**	**0.985**	**0.982**	**0.981**	**0.964**	**0.971**	**0.967**	**0.905**	**0.905**	**0.907**	*	0.027	**0.040**	**0.042**	**0.154**
GUI *L25*	**0.787**	**0.830**	**0.773**	**0.874**	**0.970**	**0.976**	**0.967**	**0.897**	**0.980**	**0.985**	**0.984**	**0.962**	**0.937**	**0.949**	**0.985**	**0.981**	**0.980**	0.957	**0.967**	**0.962**	**0.900**	**0.900**	**0.903**	0.054	*	0.009	0.028	**0.143**
SAG *L26*	**0.823**	**0.855**	**0.807**	**0.894**	**0.974**	**0.979**	**0.971**	**0.915**	**0.981**	**0.986**	**0.986**	**0.968**	**0.937**	**0.956**	**0.986**	**0.983**	**0.981**	0.966	**0.973**	**0.969**	**0.907**	**0.907**	**0.909**	0.000	0.057	*	0.007	**0.135**
CAZ *L27*	**0.782**	**0.822**	**0.772**	**0.855**	**0.925**	**0.938**	**0.934**	**0.870**	**0.946**	**0.959**	**0.958**	**0.940**	**0.937**	**0.929**	**0.959**	**0.950**	**0.946**	0.897	**0.918**	**0.907**	**0.777**	**0.777**	**0.782**	0.180	0.166	0.185	*	**0.155**
MAR *L28*	**0.823**	**0.855**	**0.807**	**0.894**	**0.974**	**0.979**	**0.971**	**0.915**	**0.981**	**0.986**	**0.986**	**0.968**	**0.937**	**0.956**	**0.986**	**0.983**	**0.981**	**0.966**	**0.973**	**0.969**	**0.907**	**0.907**	**0.909**	0.000	0.057	0.000	0.185	*
