# Supplementary material for: Phylogeography of the Spanish Moon Moth Graellsia isabellae (Lepidoptera, Saturniidae)
Source: BMC Evol Biol. 2016 Jun 24;16:139. doi: 10.1186/s12862-016-0708-y (PMC4919910; doi:10.1186/s12862-016-0708-y)

Additional file 6. Identification of the most likely number of G. isabellae groups by the analysis of microsatellite data with STRUCTURE 2.3.4.
a) Estimated log probability of data for the different number of inferred clusters (K); bars correspond to standar deviations, after 20 independent runs.
b) Rate of change in the log probability of data between successive K values (delta K).
Both figures were obtained with the aid of STRUCTURE HARVESTER 0.6.94 available at http://taylor0.biology.ucla.edu/struc_harvest/.

a)

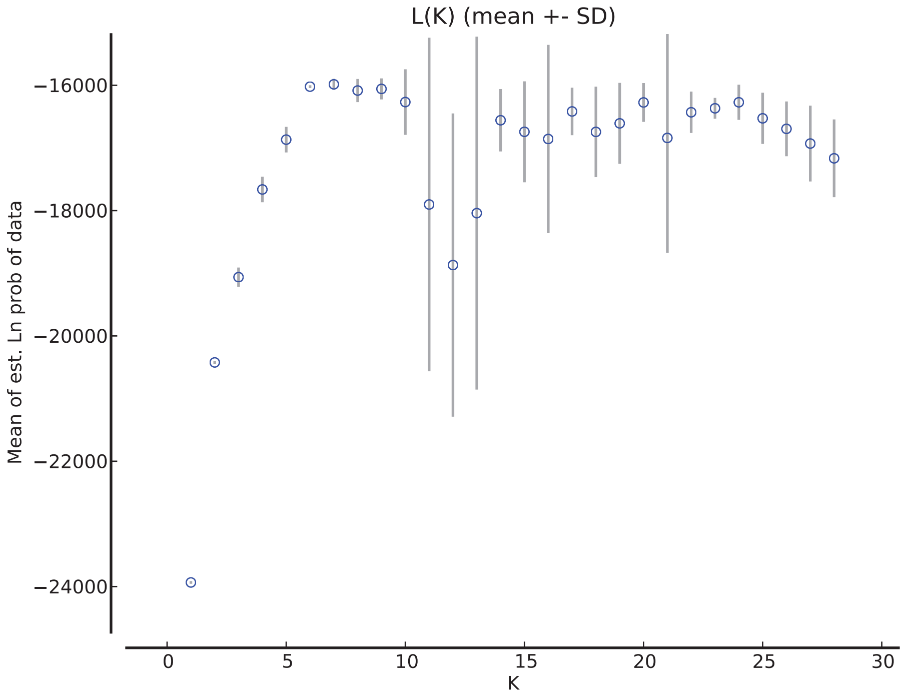

b)

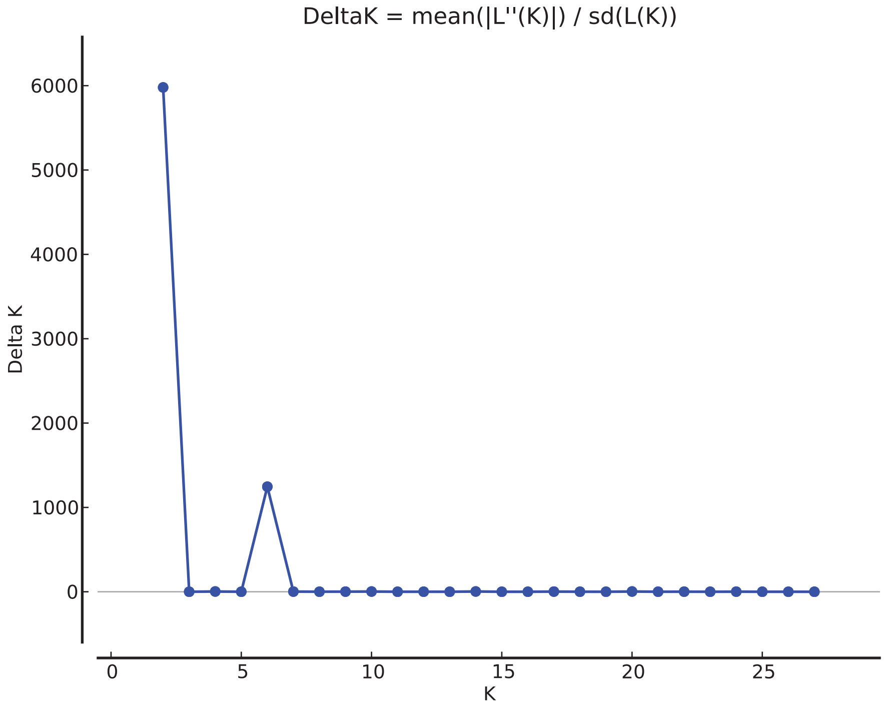

Supplement: Additional file 6; — Identification of the most likely number of G. isabellae populations by the analysis of microsatellite data with SRUCTURE 2.3.4. a) Estimated log probability of data for the different number of inferred clusters (K); bars correspond to standard deviation, after 20 independent runs; b) Rate of change in the log probability of data between successive K values (Δk). Both figures were obtained with the aid of STRUCTURE HARVESTER 0.6.94 available at http://taylor0.biology.ucla.edu/struct_harvest/. (PDF 685 kb) [file 12862_2016_708_MOESM6_ESM.pdf]
