# Supplementary material for: Phylogeography of the Spanish Moon Moth Graellsia isabellae (Lepidoptera, Saturniidae)
Source: BMC Evol Biol. 2016 Jun 24;16:139. doi: 10.1186/s12862-016-0708-y (PMC4919910; doi:10.1186/s12862-016-0708-y)
Supplement: Additional file 7: — Further details about the phylogeography of Pinus sylvestris and P. nigra and their relationship with G. isabellae. Population structure of P. sylvestris and P. nigra as revealed by different molecular markers. All figures are reproduced with kind permission of the copyright holder. (PDF 1273 kb) [file 12862_2016_708_MOESM7_ESM.pdf]

Additional file 7. Further details about the phylogeography of *Pinus sylvestris* and P. *nigra* and their relationship with *G. isabellae*.

We revised the organelle and nuclear results available for *Pinus sylvestris* and *P. nigra* in order to gain insights into the phylogeography of the two confirmed hosts of *Graellsia isabellae*, focusing on the population structure in the Iberian Peninsula. Population structure is expected to be more easily detected using data from uniparentally inherited markers. Mitochondrial DNA is maternally transmitted in Pinaceae, although rare paternal transmission has also been reported (revised by [1]). Seed dispersal distances are short, usually within a 100 m radius of the mother-tree (revised by [2]). The chloroplast is predominantly transmitted by pollen in conifers. Airborne transportation of male gametes can result in effective pollination over 100 km in *Pinus sylvestris* [3].

Floran et al. [4] defined three evolutionary units for the Scots pine: Spain, Northern/Central Europe and Northern Fennoscandia. Five works presented the variation in haplotype frequencies of 4-11 sites of the northern half of Spain: one variant [5-7] or two [8, 9] shared with other European sites.

The two forms described within *P. nigra* Arn. subsp. *salzmannii* (Dunal) (*hispanica* occurs in the Betic Mountains and Central Iberian System, *pyrenaica* is found in the Pyrenees) meet at the Eastern Iberian System (revised by [10]). Some cytogenetic traits (18rDNA and fluorochrome banding patterns) were shared between the two eastern Iberian populations representing *P. nigra salzmannii* and the Balkan *P. nigra dalmatica* [11].

The Scots pine is widely acknowledged as a Tertiary relict [4, 6]. The differentiation of *P. sylvestris* occurring at the Eastern Iberian system, sharing a mitochondrial haplotype with the Balkans [9], clearly indicates an ancestral vicariant

event. It is therefore plausible that the high diversity currently found in the Eastern Iberian cluster (EI) of *G. isabellae* is the genetic footprint of a long-standing (maybe Tertiary) historical association between *G. isabellae* and *Pinus* spp. in that area. *Pinus nigra* is known to occur in the Eastern Iberian System (near Els-Ports, *L4*) at Eemian deposits dated as 112-84 kya [12]. Preliminary data suggested that the forests of *P. nigra* from eastern Spain showed some affinity with the Balkan area [11]. Indeed, two natural populations from eastern Iberia showed the highest genetic diversity of this species within Spain [13], but more information is needed before concluding whether the Eastern Iberian system harbours also the ancestral area for the Black pine in Spain. The occurrence of ancient (Tertiary origin) populations/species in the Eastern Iberian System/Ebro Valley has already been documented for 63 species of arthropods [14], who also surveyed the parallel biogeographical pattern of certain phytophagous insects feeding on Tertiary host plant relicts in that area. Phylogeographic surveys on two of those vicariant plants also revealed their high genetic diversity in the Eastern Iberian System [15, 16].

The severe climate changes reported during the last deglaciation led the distribution range of the Scots pine to expand so that around 8 kya *P. sylvestris* had occupied its entire potential area in Europe. After 7 kya, *P. sylvestris* started to decline in the south of the Iberian Peninsula (revised by [6]). At present, the only remnants of the *P. sylvestris* forest in South Spain are two small patches, Sierra Nevada and Baza, separated by about 80 km [17], not occupied by *G. isabellae*. The mid-Holocene reconstructions by Benito Garzón et al. [18, 19] were calculated for 6 kya, when the decline of the species had already started, so that those reconstructions might be reporting a fragmentation stage after the putative SI-CI connection. Alternatively, the predicted past distributions may have underestimated the actual range of the species

due to the assumptions taken when modelling [18]. Indeed, Sierra Nevada and Baza did not appear in the Mid-Holocene reconstructions by [18], but they do in the ones by [19].

We cannot rule out *Pinus sylvestris* to have carried *G. isabellae* from SI to CI, but in that case, the source of migrants had to be Baza, rather than Sierra Nevada. Both our cpDNA results and mitochondrial markers [8] revealed the deep divergence of Sierra Nevada and the other Spanish populations of Scots pine. Our cpDNA results included Baza within the "yellow" cluster of *P. sylvestris*, which actually includes Eastern Iberian System, Pyrenees and part of the Central Iberian System, e.g. Rascafría and Cercedilla, but not Peguerinos (Figure 4a). mtDNA Haplotype frequencies were similar in Baza and some localities of the Central Iberian System [5, 7, 9]. Nuclear markers also revealed that Baza was genetically closer to the Central Iberian System than to the Eastern Iberian one [20]. These authors also highlighted the noticeable divergence between Baza and the rest of their so-called "second cluster". Therefore, the isolation of Baza from the Central Iberian System took place after the differentiation of the red (western) and yellow (eastern) groups of *P. sylvestris* (Figure 4a), which presumably date back to the LGM.

Pinus nigra could have also allowed *G. isabellae* to reach the Central Iberian System. Our reanalysis of cpDNA revealed footprints of male geneflow between the Black pine from the southernmost and central Spanish sites, the Central Iberian forest and one of the Eastern Iberian ones (Figure 4b). However, *G. isabellae* has not been found in Baza nor Huelma, but a bit north and eastwards, as for instance in Cazorla, whose Black pines grouped instead with the Iberian System. Only if *G. isabellae* were found in Sierra Nevada (a possibility revised by [21]) and/or Baza, one could get a deeper insight into the colonisation of the Central Iberian System.

References

1. Gernandt D, Willyard A, Syring J, Liston A. The Conifers (Pinophyta). In: Plomion C, Bousquet J, Kole C, editors. Genetics, Genomics and Breeding of Conifers. Enfield, NH: Science Publishers; 2011. p. 1-40.
2. Sullivan J. *Pinus sylvestris*. In: U.S. Department of Agriculture FS, Rocky Mountain Research Station, Fire Sciences Laboratory (Producer), editor. 1993.
3. Robledo-Arnuncio J. Wind pollination over mesoscale distances: an investigation with Scots pine. New Phytol. 2011;190:222-33.
4. Floran V, Sestras R, Gil M. Organelle Genetic Diversity and Phylogeography of Scots Pine (*Pinus sylvestris* L.). Not Bot Hort Agrobot Cluj. 2011;39:317-22.
5. Soranzo N, Alía R, Provan J, Powell W. Patterns of variation at a mitochondrial sequence-tagged-site locus provides new insights into the postglacial history of European *Pinus sylvestris* populations. Mol Ecol. 2000;9:1205-11.
6. Cheddadi R, Vendramin G, Litt T, Francois L, Kageyama M, Lorentz S, et al. Imprints of glacial refugia in the modern genetic diversity of *Pinus sylvestris*. Global Ecol Biogeogr. 2006;15:271-82.
7. Pyhäjärvi T, Salmela M, Savolainen O. Colonization routes of *Pinus sylvestris* inferred from distribution of mitochondrial DNA variation. Tree Genet Genomes. 2008;4:247-54.
8. Sinclair W, Morman J, Ennos R. The postglacial history of Scots pine (*Pinus sylvestris*) in western Europe: evidence from mitochondrial DNA variation. Mol Ecol. 1999;8:83-8.
9. Naydenov K, Senneville S, Beaulieu J, Tremblay F, Bousquet J. Glacial vicariance in Eurasia: mitochondrial DNA evidence from Scots pine for a complex heritage involving genetically distinct refugia at mid-northern latitudes

and in Asia Minor. BMC Evol Biol. 2007;7.

10. Bravo Oviedo A, Montero González G. Descripción de los caracteres culturales de las principales especies forestales de España. In: Serrada R, Montero M, Reque J, editors. Compendio de Selvicultura Aplicada en España. Madrid: INIA & FUCOVASA; 2008. p. 1178.

11. Bogunic F, Siljak-Yakovlev S, Muratovic E, Ballian D. Different karyotype patterns among allopatric *Pinus nigra* (Pinaceae) populations revealed by molecular cytogenetics. Plant Biol. 2011;13:194-200.

12. Roiron P, Chabal L, Figueiral I, Terral J, Ali A. Palaeobiogeography of *Pinus nigra* Arn. subsp *salzmannii* (Dunal) Franco in the north-western Mediterranean Basin: A review based on macroremains. Rev Palaeobot Palynol. 2013;194:1-11.

13. Rubio-Moraga A, Candel-Perez D, Lucas-Borja M, Tiscar P, Vinegla B, Linares J, et al. Genetic Diversity of *Pinus nigra* Arn. Populations in Southern Spain and Northern Morocco Revealed By Inter-Simple Sequence Repeat Profiles. Int J Mol Sci. 2012;13:5645-58.

14. Ribera I, Blasco-Zumeta J. Biogeographical links between steppe insects in the Monegros region (Aragon, NE Spain), the eastern Mediterranean, and central Asia. J Biogeogr. 1998;25:969-86.

15. Pérez-Collazos E, Catalán P. Genetic diversity analysis and conservation implications for the Iberian threatened populations of the irano-turanian relict *Krascheninnikovia ceratoides* (Chenopodiaceae). Biol J Linn Soc Lond. 2007;92:419-29.

16. Terrab A, Schonswetter P, Talavera S, Vela E, Stuessy T. Range-wide phylogeography of *Juniperus thurifera* L., a presumptive keystone species of western Mediterranean vegetation during cold stages of the Pleistocene. Mol

Phylogenet Evol. 2008;48:94-102.

17. Castro J, Gómez J, García D, Zamora R, Hódar J. Seed predation and dispersal in relict Scots pine forests in southern Spain. Plant Ecol. 1999;145:115-23.

18. Benito Garzón M, de Dios R, Ollero H. Predictive modelling of tree species distributions on the Iberian Peninsula during the Last Glacial Maximum and Mid-Holocene. Ecography. 2007;30:120-34.

19. Benito Garzón M, de Dios R, Ollero H. The evolution of the *Pinus sylvestris* L. area in the Iberian Peninsula from the last glacial maximum to 2100 under climate change. Holocene. 2008;18:705-14.

20. Prus-Glowacki W, Stephan B, Bujas E, Alia R, Marciniak A. Genetic differentiation of autochthonous populations of *Pinus sylvestris* (Pinaceae) from the Iberian peninsula. Plant Syst Evol. 2003;239:55-66.

21. Ibáñez Gázquez S, Nevado Ariza JC, Ylla Ullastre J. *Graellsia isabelae* (Graells, 1849), una nueva especie para la fauna lepidopterológica de Almería (España) (Lepidoptera: Saturniidae). SHILAP Soc Hispano Luso Am Lepid. 2008;36:427-30.

Population structure of a) *Pinus sylvestris* and b) *Pinus nigra* as revealed by different molecular markers.

a)

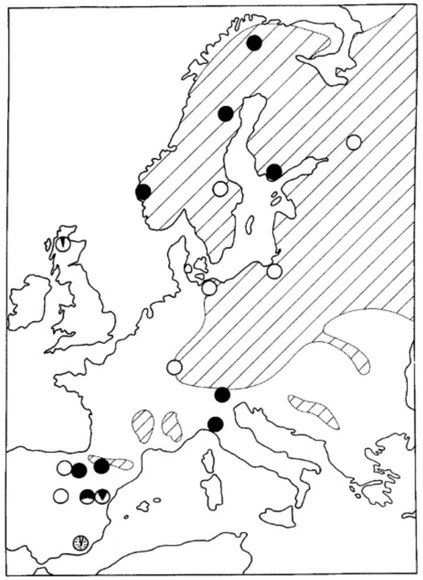

MtDNA
Figure 1 by Sinclair et al. (1999)
DOI:10.1046/j.1365-294X.1999.00527.x

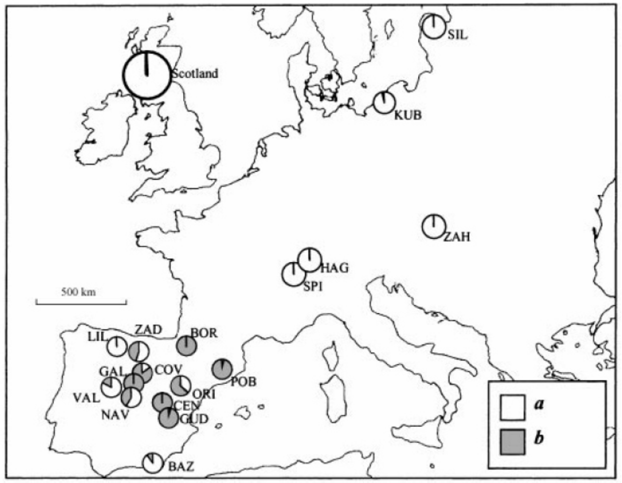

MtDNA
Figure 1 by Soranzo et al. (2000)
DOI:10.1046/j.1365-294x.2000.00994.x

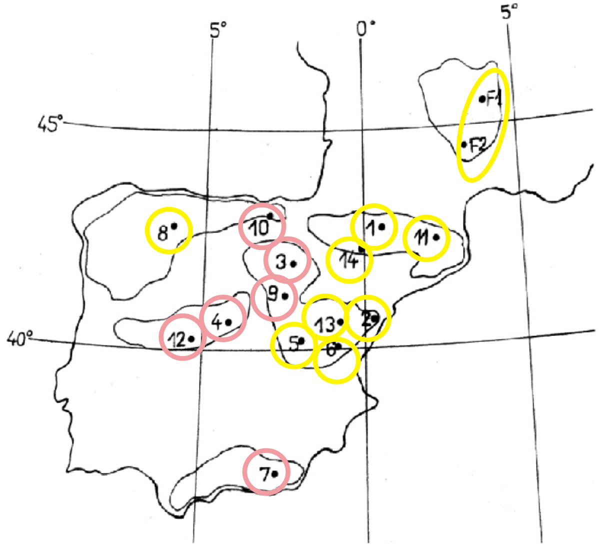

Isoenzymes
Modified from Figure 1 by Prus-Glowacki et al. (2003)
DOI:10.1007/s00606-002-0256-3

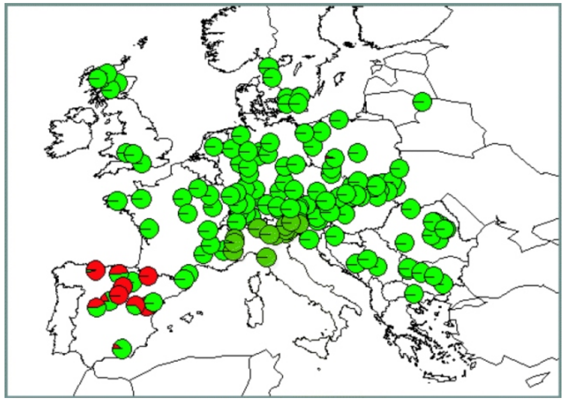

MtDNA
Figure 2 by Cheddadi et al. (2006)
DOI:10.1111/j.1466-8238.2006.00226.x

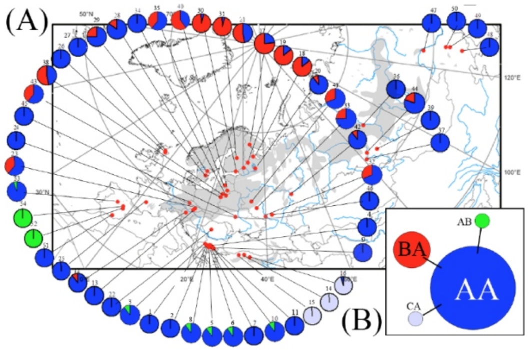

MtDNA
Figure 1 by Naydenov et al. (2007)
DOI:10.1186/1471-2148-7-233

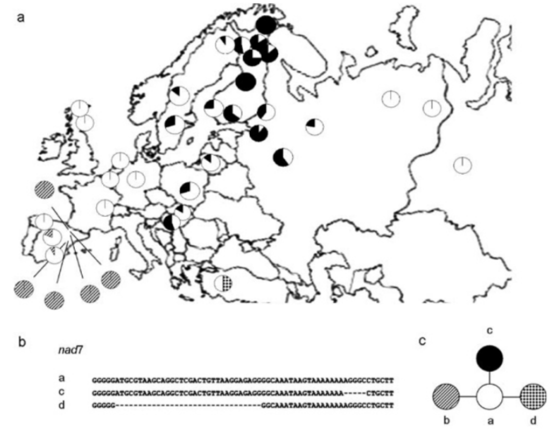

MtDNA
Figure 1 by Pyhäjärvi et al. (2008)
DOI:10.1007/s11295-007-0105-1

b)

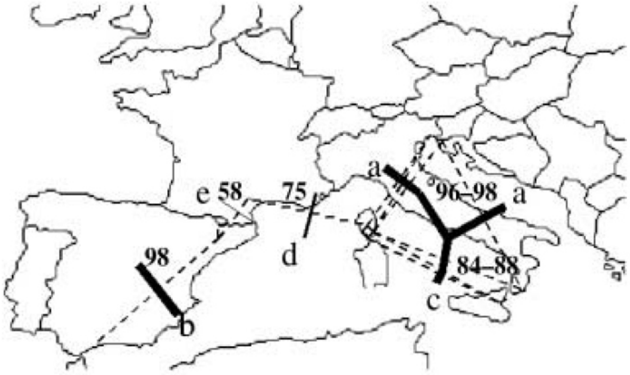

CpDNA SSR
Figure 1 by Afzal-Rafii & Dodd (2007)
DOI:10.1111/j.1365-294X.2006.03183.x

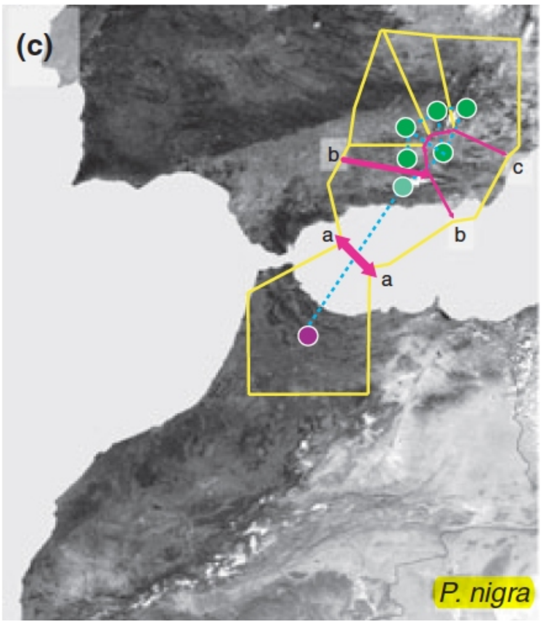

CpDNA SSR
Figure 2c by Jaramillo-Correa et al. (2010)
DOI:10.1111/j.1365-294X.2010.04912.x

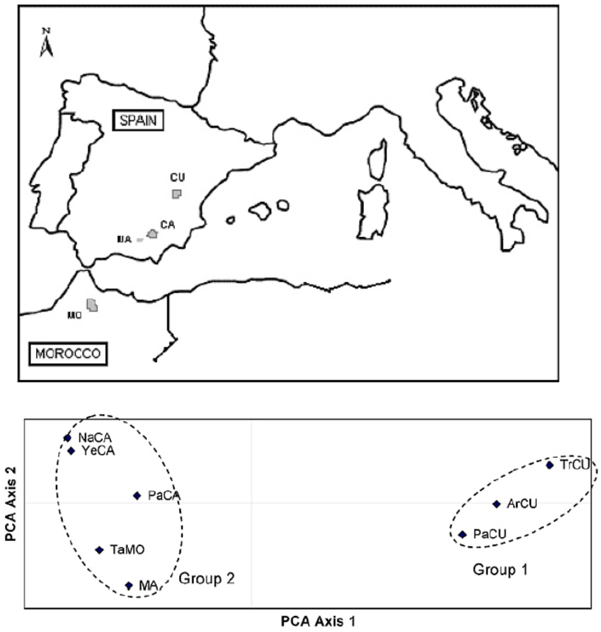

ISSR
Figures 5 and 1 by Rubio-Moraga et al. (2012)
DOI:10.3390/ijms13055645
