# Supplementary material for: Phylogeography of the Spanish Moon Moth Graellsia isabellae (Lepidoptera, Saturniidae)
Source: BMC Evol Biol. 2016 Jun 24;16:139. doi: 10.1186/s12862-016-0708-y (PMC4919910; doi:10.1186/s12862-016-0708-y)

Additional file 8. **Detailed distribution of *Pinus sylvestris* in the Spanish Pyrenees (green: natural, blue: reforested).**
a) Municipalities where samples of *Grallesia isabellae* were obtained are highlighted; b) (next page)

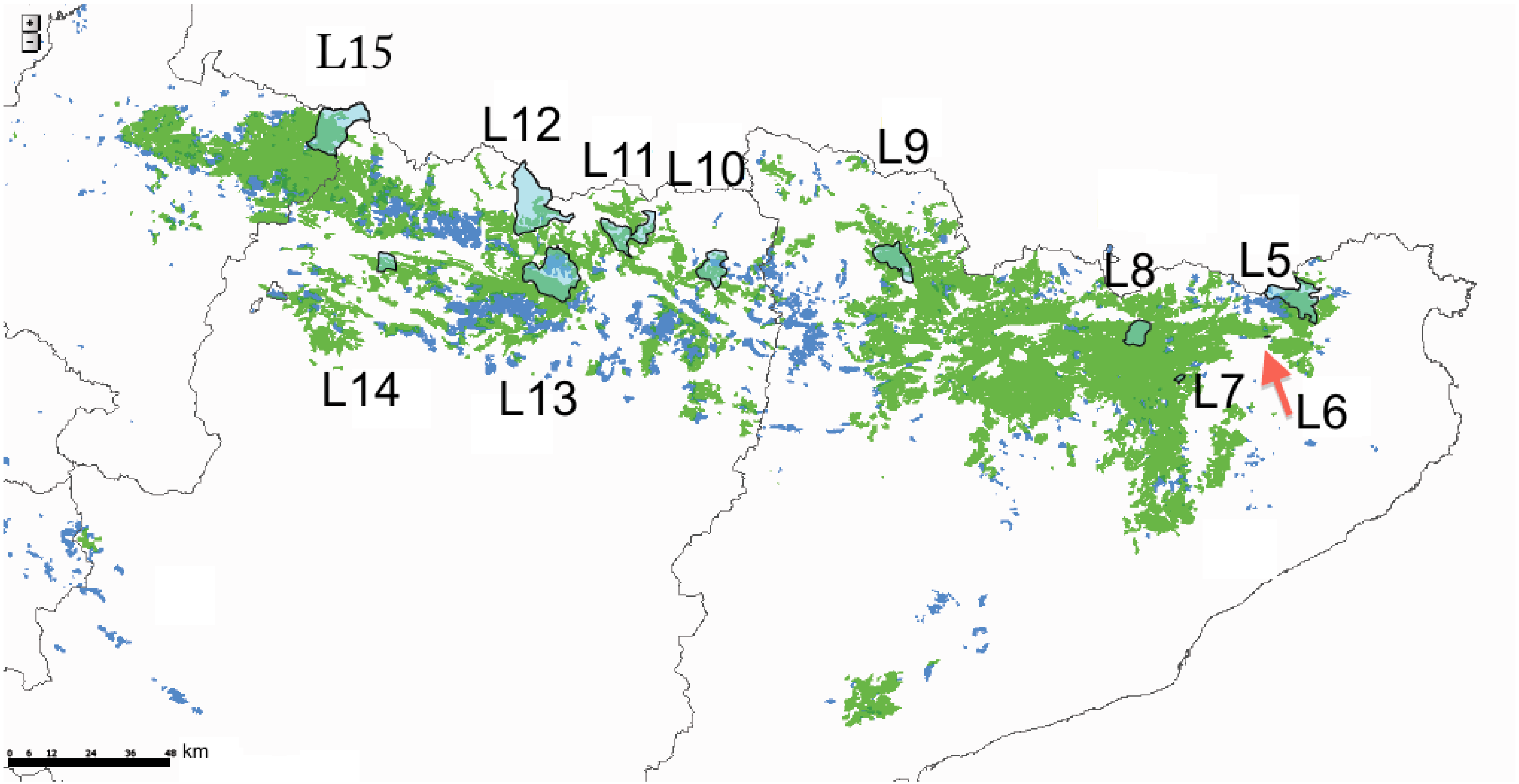

b) Distribution of *Pinus sylvestris* in the Central Pyrenees showing original (green) and reforested (blue) woodland patches as well as two relevant localities in the phylogeogrpahy of *Graellsia isabellae*: Renanué (*L10*, WP cluster, 42°29'31.7"N 0°31'42.6"E) and Baiasca (*L9*, EP cluster, 42°30'07.2"N 1°08'59.0"E) (GIS of ofrest tree species in Spain 2010).

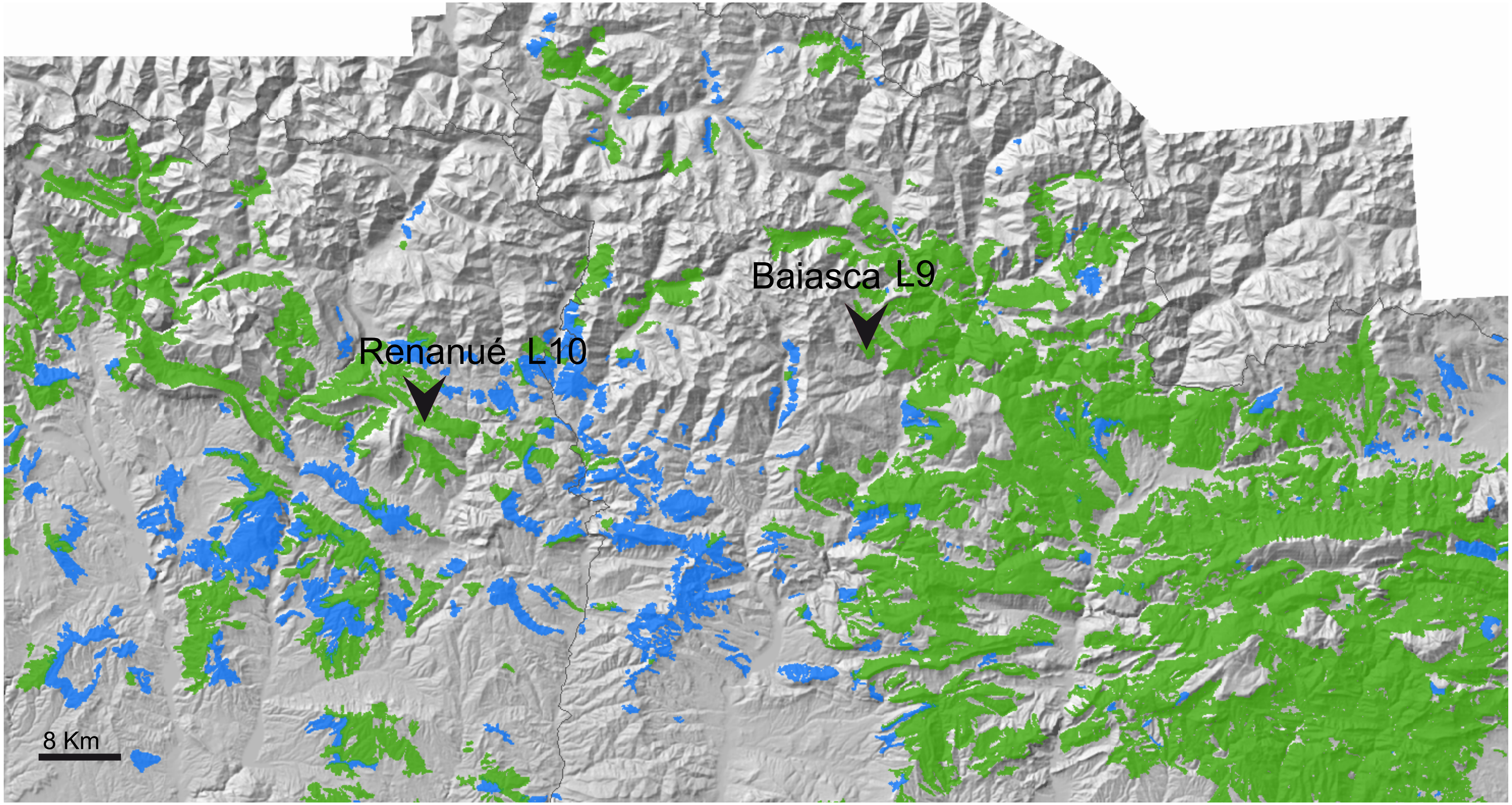

Supplement: Additional file 8: — Detailed distribution of Pinus sylvestris in the Spanish Pyrenees, both natural (green) and reforested (blue) areas. a) Municipalities where samples of G. isabellae were obtained are highlighted; b) Enlarged view of the area between Baiasca (L9) and Renanué (L10). (PDF 3010 kb) [file 12862_2016_708_MOESM8_ESM.pdf]
